# Production of chain-extended cinnamoyl compounds by overexpressing two adjacent cluster-situated LuxR regulators in *Streptomyces globisporus* C-1027

**DOI:** 10.3389/fmicb.2022.931180

**Published:** 2022-08-03

**Authors:** Xingxing Li, Weicong Ren, Yihong Li, Yuanyuan Shi, Hongmin Sun, Lifei Wang, Linzhuan Wu, Yunying Xie, Yu Du, Zhibo Jiang, Bin Hong

**Affiliations:** NHC Key Laboratory of Biotechnology of Antibiotics, CAMS Key Laboratory of Synthetic Biology for Drug Innovation, Institute of Medicinal Biotechnology, Chinese Academy of Medical Sciences & Peking Union Medical College, Beijing, China

**Keywords:** LuxR-family regulator, *ortho*-methyl phenyl alkenoic acids, cinnamoyl moiety biosynthetic gene cluster, *Streptomyces globisporus* C-1027, genome mining

## Abstract

Natural products from microorganisms are important sources for drug discovery. With the development of high-throughput sequencing technology and bioinformatics, a large amount of uncharacterized biosynthetic gene clusters (BGCs) in microorganisms have been found, which show the potential for novel natural product production. Nine BGCs containing PKS and/or NRPS in *Streptomyces globisporus* C-1027 were transcriptionally low/silent under the experimental fermentation conditions, and the products of these clusters are unknown. Thus, we tried to activate these BGCs to explore cryptic products of this strain. We constructed the cluster-situated regulator overexpressing strains which contained regulator gene(s) under the control of the constitutive promoter ermE*p in *S. globisporus* C-1027. Overexpression of regulators in cluster 26 resulted in significant transcriptional upregulation of biosynthetic genes. With the separation and identification of products from the overexpressing strain OELuxR1R2, three *ortho*-methyl phenyl alkenoic acids (compounds **1–3**) were obtained. Gene disruption showed that compounds **1** and **2** were completely abolished in the mutant GlaEKO, but were hardly affected by deletion of the genes *orf3* or *echA* in cluster 26. The type II PKS biosynthetic pathway of chain-extended cinnamoyl compounds was deduced by bioinformatics analysis. This study showed that overexpression of the two adjacent cluster-situated LuxR regulator(s) is an effective strategy to connect the orphan BGC to its products.

## Introduction

Natural products, especially secondary metabolites from microorganisms, have been widely used in the pharmaceutical industry as antibiotics, immunosuppressants, antifungal, anticancer, and anti-parasitic drugs (Hwang et al., 2014; Qin et al., 2017). Among all microorganisms, actinomycetes, especially *Streptomyces*, are well-known to be prolific producers of natural products, and about two-thirds of the known natural antibiotics are produced by *Streptomyces* (Lucas et al., 2013). The traditional programs of natural product discovery are usually based on the guidance of bioactivity screening. However, this strategy leads to the tendency of repeated discovery of known compounds, which requires new strategies to solve this problem (Nielsen et al., 2011; Gaudencio and Pereira, 2015; Patridge et al., 2016). With the rapid development of high-throughput sequencing technology, the massive microbial genomic data have revealed a potential to produce diverse and novel metabolites than previously appreciated (Choi et al., 2015; Mukherjee et al., 2017). Millions of putative biosynthetic gene clusters (BGCs) have been identified, but only a small part of them has been characterized (Rutledge and Challis, 2015). This can be attributed to the fact that most BGCs are silent or poorly expressed in their native hosts under conventional experimental fermentation conditions. On the other hand, a lot of observed natural products have not been linked to their BGCs (Hoskisson and Seipke, 2020). It is still a key challenge to discover novel natural products and link them to their BGCs.

Several approaches have been developed to activate the targeted BGCs and comprehensively reviewed recently (Rutledge and Challis, 2015; Ren et al., 2017; Baral et al., 2018; Covington et al., 2021). Based on genetic manipulation in native hosts, overexpressing putative cluster-situated positive regulators (activators) and deleting negative regulators (repressors) encoded in cryptic clusters are two technically practical and rational approaches. For instance, overexpression of a cluster-situated activator gene *chaI* led to the production of two novel chattamycins in *Streptomyces chattanoogensis* (Zhou et al., 2015). Similarly, the constitutive expression of cluster-situated activator *hcdR2* triggered the production of herbicidin in *Streptomyces mobaraensis* (Shi et al., 2019b). In contrast, deletion of *alpW*, which encodes a TetR-like cluster-situated repressor, led to the identification of a new product kinamycin in *Streptomyces ambofaciens* (Bunet et al., 2011). The cryptic jadomycin BGC could be activated by disruption of the cluster-situated repressor gene *jadR2* in *Streptomyces venezuelae* (Xu et al., 2010). Instead of genetically manipulating regulators, Wang et al. developed a transcription factor decoy strategy, which interferes with the binding of the regulator to its cognate DNA targets, and successfully activated eight silent BGCs in streptomycetes (Wang et al., 2019).

*Streptomyces globisporus* C-1027, which was isolated from a soil sample collected in the Qian-jiang area of Hubei Province, China, produces an extremely potent antitumor antibiotic lidamycin (also known as C-1027) (Hu et al., 1988; Shao and Zhen, 2008). The biosynthetic pathway and regulatory mechanism of lidamycin have been elucidated previously (Liu et al., 2002; Wang et al., 2009; Li et al., 2015). The genome sequence of this strain has been reported (Li et al., 2016), and antiSMASH (version 3.0.5) (Weber et al., 2015) analysis indicated that it contained at least 33 secondary metabolite BGCs (Supplementary Table 3), including the potential for the synthesis of compounds such as polyketides (PKs), non-ribosomal peptides (NRPs), ribosomally synthesized and post-translationally modified peptides (RiPPs), and siderophores. Although this strain has been extensively studied, it has not been found to produce secondary metabolites other than lidamycin and its precursors. To explore the ability of this strain to produce different secondary metabolites, especially PKs and NRPs with high druggability, we attempted to overexpress the transcriptional regulatory gene(s) within or near these BGCs to rapidly uncover new secondary metabolites that might be activated, before delving into the specific biosynthetic and regulatory mechanism of these understudied clusters.

Herein, we report the identification, isolation, and structure elucidation of three cinnamoyl fatty acid derivatives with a 2-methylphenyl unit by overexpression of two adjacent LuxR genes in cluster 26 of *S. globisporus* C-1027 simultaneously. This is the first time to link these chain-extended cinnamoyl fatty acids to their biosynthetic gene cluster.

## Results

### Activation of cryptic biosynthetic gene clusters in *Streptomyces globisporus* C-1027

**FIGURE 1:**
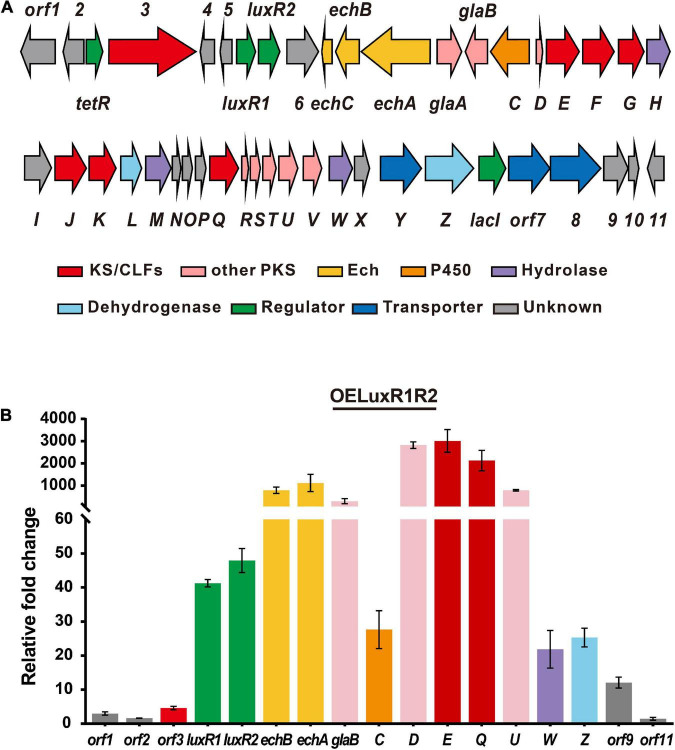
LuxR1 and LuxR2 were the cluster-specific positive regulators for cluster 26. **(A)** Organization of cluster 26. **(B)** Transcriptional analysis of cluster 26 genes in the regulator-overexpression strain detected by RT-qPCR analysis. The mycelia of regulator-overexpression strain (OELuxR1R2) were collected for the extraction of total RNAs at 48 h at the beginning of the fermentation in an FMC-1027-1 medium. These samples were subjected to RT-qPCR analysis. The relative mRNA level of the target genes was normalized to the principal sigma factor gene *hrdB*. The relative expressional level of each sample was represented as the value related to the control strain C-1027/pSET152. Values are presented as means ± SEM (three biological repeats for each strain).

Genome mining of *S. globisporus* C-1027 revealed the presence of 10 BGCs of PKs (cluster 8, 31), NRPs (cluster 7, 10, 16), or hybrid polyketide-non-ribosomal peptides (PK-NRPs, cluster 19, 26, 28, 32, 33) (Supplementary Table 3). Analysis of the transcriptome sequencing data indicates that except for the biosynthetic cluster for lidamycin (cluster 33), the other nine clusters were silent or expressed at very low levels [below 300 FPKM (fragments per kilobase of exon per million fragments mapped) value, Supplementary Figure 1]. Thus, we attempted to overexpress the putative cluster-situated regulator(s) within or near these BGCs to activate these clusters to explore uncharacterized metabolites. We constructed the putative cluster-specific regulator overexpression plasmids based on the pICLset vector under the control of the constitutively active ermE* promoter (Hong et al., 2007). Each was integrated into the chromosome of *S. globisporus* C-1027, and the transcriptional level was detected by reverse transcription-quantitative PCR (RT-qPCR). Notably, the hybrid PKS/NRPS cluster 26 contained four putative regulatory genes (*tetR*, *luxR1*, *luxR2*, and *lacI*) within or near the cluster (Figure 1A and Supplementary Table 4). Considering that *luxR1* and *luxR2* were adjacent to each other and were close to the *echA* (a peptide synthetase, NRPS) gene and the *glaE* (β-ketoacyl synthase, KS) gene, we first constructed the co-overexpression strain (OELuxR1R2). The transcriptional level of these two regulators and biosynthetic genes in cluster 26 increased significantly (Figure 1B). This result suggested that LuxR1 and/or LuxR2 might be the cluster-situated positive regulators for cluster 26. Then, four recombinant strains harboring individually overexpressed regulator gene (OETetR, OELuxR1, OELuxR2, or OELacI) were constructed. Biosynthetic genes (*echA* and *glaE*) showed much higher transcriptional levels in OELuxR1 and OELuxR2 strains than that in OETetR or OELacI strain (Supplementary Figure 2). These data further support the positive regulatory roles for LuxR1 and LuxR2 in the transcription of genes in cluster 26.

### Structural elucidation of three products from the OELuxR1R2 strain

**FIGURE 2:**
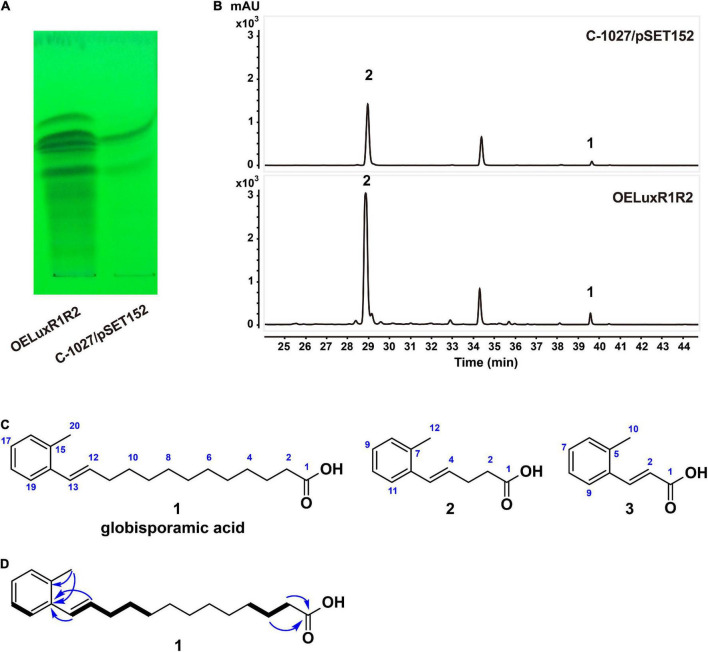
Metabolic profiles and compounds produced by recombinants. Analysis of the metabolic profiles between regulator-overexpression strain OELuxR1R2 and control strain C-1027/pSET152 by TLC **(A)** and HPLC **(B)** analysis. **(C)** Chemical structures of three compounds isolated from OELuxR1R2. **(D)** The ^1^H-^1^H COSY (thick bonds) and key HMBC correlations (arrows) of compound **1**.

As cluster 26 was increased remarkably at the transcriptional level in the OELuxR1R2 strain, we then compared the metabolic profiles of this overexpressing strain and the control strain C-1027/pSET152. Each of the fermentation broths was extracted with ethyl acetate (EtOAc) and analyzed by thin-layer chromatography (TLC, Figure 2A) or high-performance liquid chromatography (HPLC, Figure 2B). Compared with the control strain, the overexpressed strain OELuxR1R2 produced several new products by TLC analysis (Figure 2A). Moreover, the HPLC results indicated that several peaks with retention times at 28–30 and 39–40 min were significantly increased (Figure 2B). Then, the fermentation of strain OELuxR1R2 was scaled up to 20 L and the separation of target compounds was performed as described in the “Materials and Methods” section. The main components **1** and **2**, together with minor congeners **3**, were purified.

Compound **1** was obtained as a white amorphous powder and determined to have a molecular formula of C_20_H_30_O_2_ from its HRESIMS analysis (*m*/*z* 301.2174 [M-H]^–^, 0.33 ppm; Supplementary Figure 4), requiring six double-bond equivalents (DBE). Analysis of the ^1^H, ^13^C NMR, and DEPT spectra of compound **1** (Supplementary Table 5 and Supplementary Figures 5–7) returned a carbonyl (δ_*C*_ 174.9), a disubstituted benzene [δ_*C*_ 137.9, 135.6, 128.7, 127.7, 127.0, 126.3 and δ_*H*_ 7.41 (d, *J* = 7.5 Hz, 1H), 7.02 (m, 3H)], an alkene [δ_*C*_ 133.1, δ_*H*_ 6.12 (dt, *J* = 13.5, 5.5, 1H) and δ_*C*_ 131.0, δ_*H*_ 6.62 (dt, *J* = 13.5, 1.5, 1H)], a methyl [δ_*C*_ 20.0, δ_*H*_ 2.29 (s, 3H)], and 10 methylene groups [δ_*C*_ 34.4, 34.1, 30.4, 30.4, 30.3, 30.3, 30.2, 30.0, 30.0, 25.8 and δ_*H*_ 2.28 (t, *J* = 7.0 Hz, 2H), 2.25 (ddd, *J* = 13.5, 5.5, 1.5, 2H), 1.59 (m, 2H), 1.50 (m, 2H), 1.29–1.39 (m, 12H)]. A free carboxylic acid was inferred by the downfield chemical shift of the carbonyl group in compound **1** and no ester methyl or methylene signals in ^1^H and ^13^C NMR spectra. Two hydrocarbon fragments, -CH = CH-CH_2_-CH_2_-CH_2_- (F1) and -CH_2_-CH_2_-CH_2_- (F2), were indicated by the ^1^H-^1^H COSY correlations of H-13/H-12/H-11/H-10/H-9 and H-2/H-3/H-4, respectively (Figure 2D and Supplementary Figure 9). The above proposed disubstituted benzene in compound **1** was proved to be decorated by the methyl group and the alkene moiety of fragment F1, respectively, in *ortho* position according to the key HMBC correlations from the methyl protons H_3_-20 to both the *sp*^2^ quaternary carbons C-14 and C-15, and from both the alkene protons H-12 and H-13 to C-14 (Figure 2D and Supplementary Figure 10), as well as by comparison with the chemical shifts of its analog (Venugopal et al., 2002). Additionally, the free carboxylic acid was confirmed to be linked to fragment F2 according to the HMBC correlations from H-2 and H-3 to C-1 (Figure 2D and Supplementary Figure 10). Based on the above interpretation, only four methylene groups were still unassigned. Although these four methylene groups cannot be distinguished from each other due to their severely overlapped signals, it is reasonable to deduce that they are parts of the fatty acid chain and located between fragments F1 and F2 based on their nearly identical chemical shifts and by comparison with those of reported fatty acids (Gunstone et al., 1977). Therefore, compound **1** was identified as *E*-13-(2-methyl phenyl)-12-tridecenoic acid (named globisporamic acid).

Compounds **2** and **3** were identified by NMR data analysis as *E*-5-(2-methylphenyl)-4-pentenoic acid (**2**) and *E*-3-(2-methylphenyl)-acrylic acid (**3**) (Supplementary Table 5 and Supplementary Figures 11–22). Compounds **1** to **3** are all *ortho*-methyl phenyl alkenoic acids. Among them, compound **2** was previously found in a terrestrial Streptomycete GW 10/2517 (Venugopal et al., 2002) and a marine-derived *Streptomyces* isolate B8112 (Shaaban et al., 2011). *o*-Methylcinnamide (U-77863), the amide form of compound **3**, was reported in *Streptomyces griseoluteus* (Harper and Welch, 1992). During our preparation of the manuscript, the compound with the same structure as globisporamic acid was isolated from *Streptomyces* sp. QHA10 (Zhao et al., 2021). However, no related biosynthetic mechanisms have been reported for these compounds.

**FIGURE 3:**
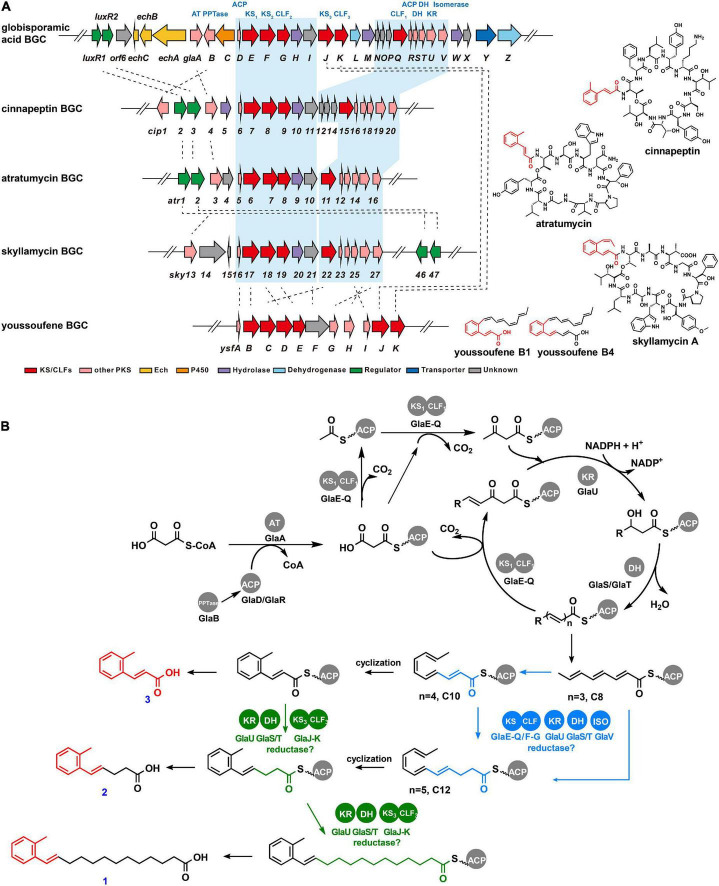
Gene cluster mining and proposed biosynthetic pathway of globisporamic acid. **(A)** Genetic organizations of the BGCs of globisporamic acid and cinnamoyl moiety-containing compounds. The blue-shaded region represented the amino acid sequence alignment of PKS genes with their homologs. The chemical structure of the natural products from the corresponding BGCs was shown on the right. **(B)** Proposed biosynthetic process of globisporamic acid. The pathway putatively catalyzed by KS_1_-CLF_1_ (GlaE-GlaQ) or KS_2_-CLF_2_ (GlaF-GlaG) was highlighted in blue, while that catalyzed by KS_3_-CLF_3_ (GlaJ-GlaK) was highlighted in green.

In particular, C-2 substituted phenyl alkenoic group known as cinnamoyl lipid moieties have been reported as part of the structure of a small family of cyclodepsipeptides, such as skyllamycin (Pohle et al., 2011), cinnapeptin (Zhang and Seyedsayamdost, 2020), atratumycin (Sun et al., 2019; Figure 3A), WS9326A (Hayashi et al., 1992), kitacinnamycin (Shi et al., 2019a), pepticinnamin E (Santa Maria et al., 2019), atrovimycin (Liu et al., 2019), and so on. It is noteworthy that the unusual *ortho*-methyl phenyl alkenoic group has only been found in atratumycin (Sun et al., 2019) and cinnapeptin (Zhang and Seyedsayamdost, 2020; Figure 3A). Among the BGCs of these compounds, the biosynthesis of cinnamoyl lipid moieties is presumed to be accomplished by type II PKS.

### Identification of the biosynthetic gene cluster for compounds 1 and 2

Cluster 26 was predicted to contain more than 40 genes by antiSMASH (version 3.0.5, Supplementary Table 4), which are encoding NRPS, type II-PKS, isomerase, dehydrogenase, hydrolase, cytochrome P450 enzyme, transporters, and regulators. The genes on the left boundary (*orf1-orf2*) and right boundary (*orf11*) might not be involved in the biosynthesis of globisporamic acid because their transcriptional levels were not as increased as that of core genes (Figure 1B).

**FIGURE 4:**
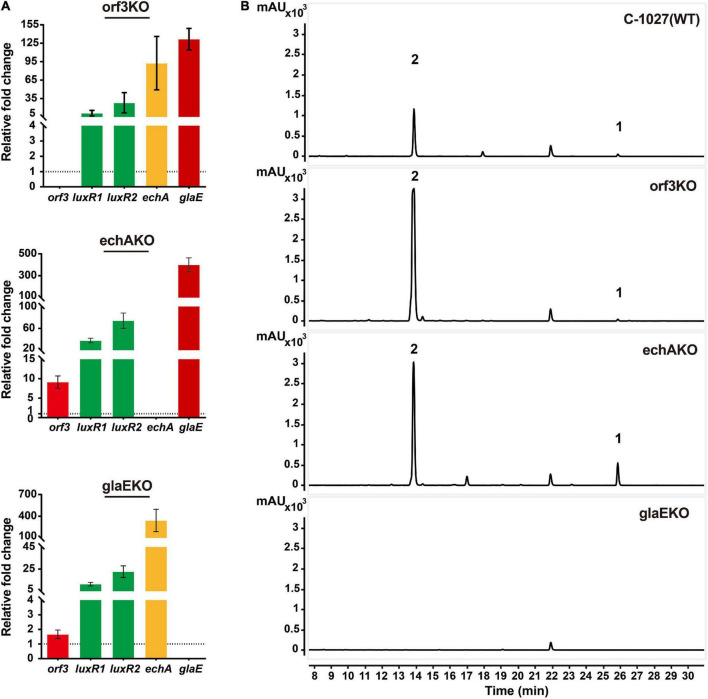
Compounds **1** and **2** were the cluster-specific products of cluster 26. **(A)** Transcriptional analysis of knockout mutants detected by RT-qPCR analysis. The relative mRNA level of the target genes was normalized to the principal sigma factor gene *hrdB*. The relative expressional level of each sample was represented as the value related to the wild-type strain *S. globisporus* C-1027. Values are presented as means ± SEM (two biological repeats for orf3KO mutant and echAKO mutant and three biological repeats for glaEKO mutant). **(B)** Analysis of the metabolic profiles of knockout mutants by HPLC-MS. The production of compounds **1** and **2** was abolished when the *glaE* gene was inactivated in the *S. globisporus* C-1027.

Since *orf3* and *glaE* were predicted as ketosynthases (KS) and the transcriptional levels of *echA* (*echABC* are homologous to biosynthetic genes for echosides) and *glaE* were both increased in OELuxR1R2 strain (Figure 1B), we constructed gene inactivation mutants to determine whether they were responsible for the biosynthesis of globisporamic acid. In the gene disrupted strains *S. globisporus* orf3KO, echAKO, and glaEKO, parts of their coding region were replaced by a thiostrepton-resistant gene (*tsr*) via gene recombination strategy and the double crossover mutants were verified by PCR (Supplementary Figures 23–25). In the corresponding mutant, the relative transcriptional level of the knockout gene was undetectable (Figure 4A). This further confirmed that the target gene was disrupted successfully. We next detected the products of the wild-type strain (C-1027) and the aforementioned three mutants by HPLC-MS analysis. It was revealed that the production of compounds **1** and **2** was completely abolished in the glaEKO strain, but was hardly affected by the deletion of *orf3* or *echA* gene (Figure 4B and Supplementary Figure 26). It was suggested that *glaE*, but not *orf3* or *echA*, was essential for the biosynthesis of compounds **1** and **2**, and these two compounds were indeed the natural products encoded by cluster 26.

Interestingly, the transcriptional levels of the regulatory genes LuxR1 and LuxR2 were increased in all three gene knockout mutants (orf3KO, echAKO, and glaEKO), and the underlying mechanism is worth further investigation. The transcriptional level of *glaE* and the yield of compounds **1** and **2** were also significantly increased in orf3KO and echAKO strains (Figure 4). The results were consistent with the improved yield of compounds **1** and **2** in the OELuxR1R2 strain (Figure 2), and further verified that LuxR1 and LuxR2 were the positive regulators for the biosynthesis of compounds **1** and **2**.

### Genome mining and proposed biosynthetic pathway of compounds 1 and 2

Genome mining revealed that GlaA-W (SGL6302-SGL6324) in cluster 26 had high sequence identities (from 35% to 77%) to type II PKSs implicated in cinnamoyl lipid moiety construction during skyllamycin (Pohle et al., 2011), cinnapeptin (Zhang and Seyedsayamdost, 2020), atratumycin (Sun et al., 2019), and youssoufenes (Deng et al., 2021) biosynthesis (Figure 3A and Supplementary Table 4). Unlike the canonical type II PKS, the PKS system involved in cinnamoyl lipid biosynthesis was phylogenetically different (Supplementary Figure 27; Shi et al., 2019a; Deng et al., 2021), which generally contained two or three pairs of ketosynthase/chain length factor (KS/CLF) complexes and harboring an isomerase-dependent polyunsaturated chain elongation process (Deng et al., 2021). In particular, there were three pairs of KS-CLF (GlaE-GlaQ, GlaF-GlaG, and GlaJ-GlaK) complexes in the *gla* cluster, similar to that of youssoufene’s BGC (*ysf*). The three *ysf* KS-CLFs were deciphered to modularly assemble different parts of the youssoufene skeleton. YsfB-YsfC initiated chain elongation, and then YsfD-YsfE yield the short-chain youssoufenes (C_18_), followed by YsfJ-YsfK to generate the long-chain youssoufenes (C_20_, C_22_). Of note, GlaJ-GlaK exhibits homology to YsfJ-YsfK (Supplementary Figure 27), which recognizes ACP-linked benzene ring containing intermediates instead of acyclic polyene intermediates in youssoufene’s biosynthesis. It is tempting to speculate that GlaJ-GlaK may have similar catalytic characteristics. GlaV showed 47% identity to the glutathione-dependent *cis–trans* isomerase YsfG of youssoufene’s cluster, suggesting that it may possess the ability to transform configuration for cyclization and participate in the chain elongation process.

Therefore, a biosynthetic pathway for compounds **1** and **2** can be proposed based on bioinformatic analysis of the *gla* cluster (Figure 3B). To begin with, a phosphopantetheinyl transferase (PPTase, GlaB) post-translationally transfers the pantetheine prosthetic group to convert *apo*-acyl carrier protein (ACP, GlaD/GlaR) into its activated form, *holo*-ACP. Initially, malonyl-CoA, as the basic building block, is loaded onto the *holo*-ACP via acyltransferase (AT, GlaA) to obtain the malonyl-ACP. The acetyl-ACP is generated by decarboxylation of malonyl-ACP and is condensed with malonyl-ACP by the catalysis of KS_1_-CLF_1_ (GlaE-GlaQ) to form the acetoacetyl-ACP. The chain elongation process carries out a repeating cycle of condensation, reduction, and dehydration, which were catalyzed by KS_1_-CLF_1_ (GlaE-GlaQ), ketoreductase (KR, GlaU), and dehydratase (DH, GlaS/GlaT), respectively, to generate a polyene with three carbon–carbon double bonds (*n* = 3, C_8_). Then, the polyene precursor was transformed from *trans-* to *cis*-configuration by isomerase (GlaV), bringing the carbon atoms within the required distance for cyclization. As it remains unclear whether the putative different length polyene precursor for compounds **1**, **2**, and **3** (C_20_, C_12_, and C_10_) was generated after the benzene ring closure, we consequently speculated two pathways (Figure 3B), and the difference is that the chain elongation of polyene precursors was catalyzed by KS_1_-CLF_1_ (GlaE-GlaQ) and KS_2_-CLF_2_ (GlaF-GlaG) (blue pathway), or catalyzed by KS_3_-CLF_3_ (GlaJ-GlaK) (green pathway), as KS_3_-CLF_3_ was proposed to participate in the chain elongation of benzene ring containing intermediates.

Like other cinnamoyl moiety-containing clusters, the enoyl-ACP reductase (ER), involved in further reduction of the double bond to a saturated intermediate, is absent in the *gla* cluster. In addition, the functions of some modified enzymes, including cytochrome P450 enzyme, dehydrogenase, and hydrolase, need to be further investigated. Moreover, the homologs of LuxR1 and LuxR2 also appeared in skyllamycin (Pohle et al., 2011), cinnapeptin (Zhang and Seyedsayamdost, 2020), and atratumycin (Sun et al., 2019) BGCs (Supplementary Table 4 and Supplementary Figure 28). Given that LuxR1 and LuxR2 have been identified as positive regulators in cluster 26, we hypothesized that they might be conservative cluster-situated regulators for cinnamoyl moiety-containing natural products biosynthesis.

### Biological activity test of compounds 1 and 2

The cinnamoyl moiety containing depsipeptides exhibited a broad range of biological activities, for example, atratumycin and atrovimycin exerted specific activity against *Mycobacteria tuberculosis* (Liu et al., 2019; Sun et al., 2019). The benzoic polyene acid youssoufene A1 was moderately active against multidrug-resistant *Enterococcus faecalis* (Li et al., 2020). However, both compounds **1** and **2** failed to display antibacterial activities at the concentration of 1,024 μg/mL against *Pseudomonas aeruginosa*, *Mycobacterium smegmatis*, *Staphylococcus aureus*, *Escherichia coli*, *E. coli*Δ*tolC* mutant, and *Bacillus subtilis*. Also, no antiviral activities were observed at the concentration of 500 μg/mL of compound **1** or **2** against coxsackievirus B3, herpes simplex virus type 1, and influenza A virus (H3N2). Zhao et al. (2021) showed compound **1** had potential activity to inhibit nitric oxide (NO) production in lipopolysaccharide (LPS)-induced mouse macrophages. Their biological activity is worthy of further exploration.

## Discussion

The genome of *Streptomyces* has the potential to encode a variety of natural metabolites. One of the challenges in natural product discovery is that the majority of BGCs of the natural product have not been characterized, partially because they are either transcriptionally silent or expressed at a very low level under standard laboratory conditions. BGCs in streptomycete often contain at least one ultimate positive transcriptional regulator for the activation of the cluster, and sometimes contain negative regulators as well. Not surprisingly, increasing positive or decreasing negative cluster-situated regulatory gene expression levels were the effective method for improving secondary metabolite titers (Rutledge and Challis, 2015). Since the genetic manipulation of overexpression regulatory genes in *Streptomyces* is far more convenient than gene knockout, we tried to overexpress the predicted regulatory genes situated in the clusters to rapidly discover novel secondary metabolites without deciphering their positive or negative regulatory mechanisms. Applying this strategy to nine cryptic clusters of PKS, NRPS, or PKS/NRPS (usually encoding compounds with high druggability) in *S. globisporus* C-1027, we significantly upregulated the transcription of cluster 26, and isolated and identified its products, including globisporamic acid and two congeners compounds **2** and **3**. We also linked the structures of the compounds to their biosynthetic gene cluster.

LuxR-family proteins have often been found as a class of cluster-situated transcriptional regulators that are widespread in actinomycetes’ secondary metabolism BGCs. Examples include HcdR2 (from herbicidin BGC in *S. mobaraensis*) (Shi et al., 2019b), GdmRI and GdmRII (from geldanamycin BGC in *S. hygroscopicus*) (He et al., 2008), PikD (from pikromycin in *S. venezuelae*) (Wilson et al., 2001), PimM (from pimaricin in *S. natalensis*) (Anton et al., 2007), PteF (from filipin in *S. avermitilis*) (Vicente et al., 2014), Tcp29 (Tei16*, from teicoplanin in *Actinoplanes teichomyceticus*) (Horbal et al., 2014), and so on. Herein, LuxR1 (SGL6295) and LuxR2 (SGL6296), two LuxR family regulators encoded within the *gla* cluster of *S. globisporus* C-1027, are demonstrated to positively regulate the biosynthesis of compounds **1** and **2**, especially when overexpressed simultaneously. Genome mining suggested that the highly conserved homologs of LuxR1 and LuxR2 were also present in the BGCs for some known cinnamoyl moiety-containing natural products, such as skyllamycin (Pohle et al., 2011), cinnapeptin (Zhang and Seyedsayamdost, 2020), and atratumycin (Sun et al., 2019; Figure 3A and Supplementary Table 4). Noteworthy, these LuxRs in the BGCs of cinnamoyl moiety-containing natural products, especially two adjacent LuxRs in the same cluster, tended to group together in the phylogenetic tree (Supplementary Figure 28). Thus, we speculated that overexpression of the adjacent LuxR-family regulators may be used to explore the orphan BGCs of novel cinnamoyl moiety-containing natural products discovered by genome mining.

In this study, three cinnamoyl fatty acid analogs were isolated and structurally elucidated from the recombinant strain OELuxR1R2. They all contained an *ortho*-methylphenyl unit with different lengths of the alkenoic acid side chain. Among them, compound **1**, globisporamic acid, contains a unique long-chain composed of 13 carbons, while compounds **2** and **3** contain a shorter side chain. Compound **3** is *o*-methyl cinnamic acid with an acrylic side chain. The biosynthesis of these congeners was demonstrated by gene knockout to be catalyzed by type II PKS, and we speculate that the chain length is determined by three pairs of KS-CLF complexes as in the biosynthesis of youssoufene. The KS-CLFs encoded in *gla* cluster along with reductase and dehydratase were proposed to catalyze the formation of different length polyene precursors, and the detailed mechanism needs to be further investigated.

Different from several reported cinnamoyl lipid-containing compounds [such as skyllamycin (Pohle et al., 2011), cinnapeptin (Zhang and Seyedsayamdost, 2020), and atratumycin (Sun et al., 2019)], which contain cyclic depsipeptide assembled by NRPS, the structure of the products of cluster 26 in *S. globisporus* C-1027 was much simpler. The only NRPS in cluster 26 was EchA (SGL6301). BLASTP alignment showed that SGL6301, SGL6299, and SGL6300 had high similarity with EchA, EchB, and EchC, which are involved in the biosynthesis of echosides, a class of *para*-terphenyl natural products isolated from *Streptomyces* sp. LZ35 (Zhu et al., 2014). Through the adenylation (A) domain sequence analysis (Rottig et al., 2011), 10 active-site residues, which are responsible for amino acid substrate specificity, were identified as VAEFVGAAGK and the substrate recognized by SGL6301 should be the same as that of EchA for echosides. The T and TE domains also possessed the established signature active motifs (LGGTSL and GYSFG, respectively) (Schwarzer et al., 2003; Zhu et al., 2014). These suggested that the product of the *ech* cassette in cluster 26 should be echoside’s congener. Since *ech* cassette and PKS core genes in cluster 26 were adjacent to each other and the transcriptional levels were both upregulated in the regulator overexpression strain OELuxR1R2, it was speculated that they might synergistically synthesize an NRPS-like product containing cinnamoyl moiety. However, neither echoside’s congener nor possible hybrid NRPS-PKS product has been found under our current fermentation and isolation conditions. This is worthy of further exploration.

## Conclusion

In summary, we activated the *gla* cluster by simultaneous overexpression of two adjacent cluster-situated positive LuxR regulator(s) in *S. globisporus* C-1027. Three *ortho*-methyl phenyl alkenoic acids were isolated and identified from the recombinant strain OELuxR1R2. Its biosynthetic pathway was proposed according to *in vivo* gene inactivation and the bioinformatic analysis of the type II PKS assembly line. This strategy can be easily and rapidly applied to the exploration of much more of such orphan gene clusters present in actinomycete genomes.

## Materials and methods

### Strains, plasmids, and growth conditions

*Streptomyces globisporus* C-1027 and its derivatives were grown at 28°C on S5 agar (Wang et al., 2009) for sporulation and in an FMC-1027-1 medium (Wang et al., 2009) for fermentation. Mannitol soya flour (MS) agar (Kieser et al., 2000) was used for conjugation. Trypticase Soy Broth (TSB, BD Difco, Sparks, MD, United States) was used for the isolation of genomic DNA. *E. coli* DH5α (Sambrook, 2001) was used as a host for cloning experiments. *E. coli* ET12567/pUZ8002 (Kieser et al., 2000) was used for conjugal transfer. *E. coli* strains were grown in liquid or solid Luria-Bertani (LB) medium at 37°C. When required, strains were incubated with apramycin (Am, 50 μg/mL), ampicillin (Amp, 100 μg/mL), kanamycin (Km, 50 μg/mL), thiostrepton (Th, 30 μg/mL), and chloramphenicol (Cm, 25 μg/mL). The bacterial strains and plasmids used in this study are listed in Supplementary Table 1.

### Transcriptome sequencing analysis

Strains were collected for the extraction of total RNAs at 48 h at the beginning of the fermentation in an FMC-1027-1 medium (Li et al., 2015). Total RNAs were isolated using the TRIzol reagent (Invitrogen, Carlsbad, CA, United States) and chloroform followed by a PureLink RNA Mini Kit (Invitrogen, Carlsbad, CA, United States) according to the manufacturer’s instructions. Samples were treated with DNase I to eliminate the genomic DNA. The quantity and quality of RNA samples were assessed as described previously (Uguru et al., 2005).

cDNA library construction and Illumina sequencing were performed by Beijing Genomics Institute (Shenzhen, China). The sequencing reads were aligned to the *S. globisporus* C-1027 reference genome. The distribution of the transcriptome reads was shown in the Integrated Genome Browser (Nicol et al., 2009). The gene expression quantifications were estimated using FPKM (fragments per kilobase of exon per million fragments mapped) values by the RSEM tool (Li and Dewey, 2011).

### Regulator overexpression in *Streptomyces globisporus* C-1027

The vector pL646 (Hong et al., 2007), a pSET152 (Bierman et al., 1992)-derived expression plasmid with the strong constitutive promoter ermE*p, was used for gene overexpression. The coding region of regulators was amplified from *S. globisporus* C-1027 genomic DNA by PCR with specific primers (Supplementary Table 2). The amplified DNA fragments were cloned into *Nde*I - *Bam*HI or *Nde*I - *Xba*I sites of pL646 to obtain the overexpression of plasmids. The recombinant vectors and control plasmid pSET152 (Bierman et al., 1992) were introduced into *S. globisporus* C-1027 by intergeneric conjugation from *E. coli* ET12567/pUZ8002 according to the established protocol (Li et al., 2015).

### Reverse transcription-quantitative PCR analysis

*Streptomyces globisporus* C-1027 or its derivative strains were collected at 48 h at the beginning of the fermentation in an FMC-1027-1 medium (Li et al., 2015). Total RNAs were isolated as described above. The first-strand synthesis of cDNA was performed with the TransScript One-Step gDNA Removal and cDNA Synthesis SuperMix (TransGen, Beijing, China) using random primers according to the manufacturer’s instructions. Primers were designed to amplify fragments of about 100 to 180 bp from the target genes (Supplementary Table 2). Quantitative PCR (qPCR) reaction was performed as described previously (Wang et al., 2009). Two qPCR replicates were performed for each cDNA, and the relative cDNA level of target genes was normalized to the level of *hrdB* according to Pfaffl’s method (Pfaffl, 2001).

### Analysis of products by thin-layer chromatography, high-performance liquid chromatography, or high performance liquid chromatography mass spectrometry

*Streptomyces globisporus* C-1027 or its derivative strains were cultured in 50 mL TSB medium at 28°C for 2 days, and then 10% culture was transferred into 100 mL FMC-1027-1 medium for continuous fermentation at 28°C for 5 days. Each fermented supernatant was extracted three times with an equal volume of ethyl acetate (EtOAc). The organic phase was combined and vacuum-dried and then dissolved with 2 mL acetone or methanol. The extract was analyzed by TLC, HPLC, or HPLC-MS.

In the silica gel TLC, EtOAc extract was separated using a mixture of solvent system with chloroform:methanol:acetic acid 100:20:1, v/v/v. The dark bands were observed under ultraviolet light at a λ of 254 nm.

High-performance liquid chromatography was performed on an Agilent 1100 HPLC using a Waters XBridge C18 column (4.6 mm × 150 mm, 3.5 μm) in a two-phase system, with phase A for methanol and phase B for 0.1% formic acid–water solution. The optimized program included column elution with a linear gradient of A/B = 10/90–100/0, v/v, over 40 min; flow rate = 0.8 mL/min, λ = 254 nm.

High-performance liquid chromatography mass spectrometry analysis was performed on an Agilent 1100 HPLC coupled to Agilent 6410 Triple Quadrupole mass spectrometer equipped with an electrospray ionization source. Chromatographic separation was performed using a Waters XBridge C18 column (4.6 mm × 150 mm, 3.5 μm) in a two-phase system, with phase A for ACN and phase B for 0.1% formic acid–water solution. The optimized program included column elution with a linear gradient of A/B = 15/85 – 100/0, v/v, over 30 min; flow rate = 1.0 mL/min, λ = 254 nm. MS spectral data were collected in the negative-ion mode, with a mass range of *m/z* 100 to 1,000. The capillary voltage was set to 3,500 V and fragmentor voltage was 120 V. The desolvation temperature was 350°C, with a desolvation gas flow of 11 L/min and a nebulizer pressure of 50 psi.

### Isolation and characterization of compounds 1–3

Fermentation supernatant (20 L) was extracted two times with an equal volume of EtOAc. The combined organic phases were evaporated under reduced pressure and mixed with 1.1 g silica gel and then separated by silica column chromatography using chloroform:methanol [100:0, v/v, (0.2 g, Fr-1); 50:1, v/v, (0.1 g, Fr-2); 10:1, v/v, (0.12 g, Fr-3); 5:1, v/v, (0.15 g, Fr-4); and 1:1, v/v, (0.12 g, Fr-5)] to obtain the five fractions. TLC analysis was carried out using chloroform:methanol:acetic acid (100:20:1, v/v/v), and the target compound was found to be distributed in Fr-2 and Fr-3 fractions. Fr-2 and Fr-3 were merged and separated by preparation TLC [developing solvent as chloroform:methanol:acetic acid (100:10:1, v/v/v)]. Two darker bands on the TLC were collected, and the silicon powder was eluted with chloroform. The eluted product was evaporated and further purified by semi-preparative HPLC using different concentrations of ACN–water solution to obtain compounds **1–2**: compound **1** (35 mg, 95% ACN–water solution); compound **2** (20 mg, 80% ACN–water solution). Fr-4 (0.15 g) was a pale-yellow oily substance, and a color band (chloroform:methanol:acetic acid 50:10:1, v/v/v) was separated by preparation TLC. After semi-preparation and HPLC purification, compound **3** (20.5 mg) was obtained.

### Construction of gene knockout mutants

Genes *glaE* (*SGL6306*), *echA* (*SGL6301*), and *orf3* (*SGL6292*) were inactivated by substituting each of the core regions with a thiostrepton resistance gene via homologous recombination (Supplementary Figures 23–25). The primers (Supplementary Table 2) for *glaE* disruption introduced restriction sites into the arms (*Hin*dIII and *Xba*I in the upstream arm, and *Xba*I and *Bam*HI in the downstream arm). The amplified flanking DNA fragments were ligated into the pUC119 vector to give pUC-LR. Then, the thiostrepton resistance gene was amplified from pIJ680 and was cloned into the *Xba*I sites of the pUC-LR vector to yield pUC-LtR. The disruption box of *glaE* was ligated into plasmid pKC1139 to give pKC-LtR. Following the transformation into *E. coli* ET12567/pUZ8002, disruption plasmid pKC-LtR was conjugated into *S. globisporus* C-1027. Recombinant strains were selected for thiostrepton resistance, and correct knockout recombinants were confirmed by PCR (Supplementary Figure 25). With the same strategy, knockout mutants for *echA* and *orf3* genes were selected on MS agar containing thiostrepton resistance and confirmed by PCR (Supplementary Figures 23, 24).

### Antibacterial activity test

The *in vitro* antibacterial activity of compounds against *Pseudomonas aeruginosa* 11, *Mycobacterium smegmatis* CPCC 240556, *Staphylococcus aureus* CPCC 100051, *Escherichia coli* O11, *E. coli*Δ*tolC* mutant, and *Bacillus subtilis* CMCC(B) 63501 were determined through the broth microdilution method. The bacteria (5 × 10^5^ per well) were grown in Mueller–Hinton broth (Oxoid) in a 96-well plate at 37°C. Compounds were tested at a concentration range of two-fold dilution from 1,024 μg/mL to 8 μg/mL. Streptomycin was tested at a concentration range of two-fold dilution from 64 to 0.5 μg/mL and used as a positive control. DMSO (2%) was used as a negative control. The minimum inhibitory concentration (MIC) was defined as the lowest concentration of compound that prevents turbidity after 16 h incubation at 37°C. The experiment was performed in duplicate.

### Antiviral activity test

The antiviral activity of compounds against coxsackievirus B3, herpes simplex virus type 1, and influenza A virus (H3N2) were performed as described previously (Ji et al., 2010; Ma et al., 2013). The 50% cytotoxic concentration (TC_50_) and the 50% inhibitory concentration (IC_50_) of the test samples were calculated using the Reed–Muench method (Pizzi, 1950).

### Bioinformatics analysis

Secondary metabolite biosynthesis gene clusters were predicted by the online resource antiSMASH^1^. PCR and qPCR primers were designed with the online resource primer3web^2^. BLASTP was used for genome mining of potential cinnamoyl moiety-containing natural products’ clusters. Amino acid sequence similarity of protein homologous was retrieved using Blastp tools. The phylogenetic tree was calculated using the MEGA-X program based on the Neighbor-joining method (Kumar et al., 2018).

## Data availability statement

The datasets presented in this study can be found in online repositories. The names of the repository/repositories and accession number can be found below: The RNA-sequencing data is publicly available at NCBI’s repository under BioProject ID: RJNA854280, (https://www.ncbi.nlm.nih.gov/bioproject/PRJNA854280), and SRA ID: SRR19905466. The HPLC-MS raw data have been deposited in the EMBL-EBI MetaboLights database (https://www.ebi.ac.uk/metabolights/) with the identifier MTBLS5152. The HPLC-MS data will be released to public on 21-July according to the database’s policies.

## Author contributions

XL, WR, and ZJ performed most of the experiments, analyzed the primary data, and wrote the draft manuscript. YL, YS, HS, LFW, LZW, YX, and YD assisted the microbiologic and chemical work in this study. BH supervised the whole research work and revised the manuscript extensively. All authors have read and approved the final manuscript.

## Footnotes

1
http://antismash.secondarymetrics.org

2
https://primer3.ut.ee
