# Supplementary material for: Production of chain-extended cinnamoyl compounds by overexpressing two adjacent cluster-situated LuxR regulators in Streptomyces globisporus C-1027
Source: Front Microbiol. 2022 Aug 3;13:931180. doi: 10.3389/fmicb.2022.931180 (PMC9381841; doi:10.3389/fmicb.2022.931180)
Supplement: Supplementary file 1 [file Data_Sheet_1.docx]

**Supplementary material**

**Table of Contents**

[Table S1. Strains and plasmids used in this study. 3](#_Toc107522077)

[Table S2. PCR primers used in this study. 5](#_Toc107522078)

[Table S3. Predicted Secondary metabolite biosynthetic gene clusters in *S. globisporus* C-1027 by antiSMASH. 8](#_Toc107522079)

[Table S4. Annotation and predicted function of genes in cluster 26. 9](#_Toc107522080)

[Table S5. ^1^H and ^13^C NMR data for compounds 1-3 in (CD_3_)_2_CO. 13](#_Toc107522081)

[Figure S1. Transcriptional analysis of secondary metabolites biosynthetic gene clusters in *S. globisporus* C-1027. 15](#_Toc107522082)

[Figure S2. Expression levels of cluster 26 genes in regulator-overexpression strains detected by RT-qPCR analysis. 16](#_Toc107522083)

[Figure S3. (-)-ESI-MS of compound 1. 17](#_Toc107522084)

[Figure S4. (-)-ESI-HRMS analysis of compound 1. 17](#_Toc107522085)

[Figure S5. ^1^H-NMR spectrum of compound **1** (500 MHz, (CD_3_)_2_CO). 18](#_Toc107522086)

[Figure S6. ^13^C-NMR spectrum of compound **1** (125 MHz, (CD_3_)_2_CO). 19](#_Toc107522087)

[Figure S7. DEPT spectrum of compound **1** (125 MHz, (CD_3_)_2_CO). 20](#_Toc107522088)

[Figure S8. HSQC spectrum of compound **1** ((CD_3_)_2_CO). 21](#_Toc107522089)

[Figure S9. ^1^H-^1^H COSY spectrum of compound **1** ((CD_3_)_2_CO). 22](#_Toc107522090)

[Figure S10. HMBC spectrum of compound **1** ((CD_3_)_2_CO). 23](#_Toc107522091)

[Figure S11. ^1^H-NMR spectrum of compound **2** (500 MHz, (CD_3_)_2_CO). 24](#_Toc107522092)

[Figure S12. ^13^C-NMR spectrum of compound **2** (125 MHz, (CD_3_)_2_CO). 25](#_Toc107522093)

[Figure S13. DEPT spectrum of compound **2** (125 MHz, (CD_3_)_2_CO). 26](#_Toc107522094)

[Figure S14. ^1^H-^1^H COSY spectrum of compound **2** ((CD_3_)_2_CO). 27](#_Toc107522095)

[Figure S15. HSQC spectrum of compound **2** ((CD_3_)_2_CO). 28](#_Toc107522096)

[Figure S16. HMBC spectrum of compound **2** ((CD_3_)_2_CO). 29](#_Toc107522097)

[Figure S17. ^1^H-NMR spectrum of compound **3** (500 MHz, (CD_3_)_2_CO). 30](#_Toc107522098)

[Figure S18. ^13^C-NMR spectrum of compound **3** (125 MHz, (CD_3_)_2_CO). 31](#_Toc107522099)

[Figure S19. DEPT spectrum of compound **3** (125 MHz, (CD_3_)_2_CO). 32](#_Toc107522100)

[Figure S20. ^1^H-^1^H COSY spectrum of compound **3** ((CD_3_)_2_CO). 33](#_Toc107522101)

[Figure S21. HSQC spectrum of compound **3** ((CD_3_)_2_CO). 34](#_Toc107522102)

[Figure S22. HMBC spectrum of compound **3** ((CD_3_)_2_CO). 35](#_Toc107522103)

[Figure S23. Disruption of *orf3* gene in *S. globisporus* C-1027. 36](#_Toc107522104)

[Figure S24. Disruption of *echA* gene in *S. globisporus* C-1027. 37](#_Toc107522105)

[Figure S25. Disruption of *glaE* gene in *S. globisporus* C-1027. 38](#_Toc107522106)

[Figure S26. EIC traces of three knockout mutants. 39](#_Toc107522107)

[Figure S27. The phylogenetic trees for KSs and CLFs. 40](#_Toc107522108)

[Figure S28. The phylogenetic tree for LuxR-family regulators. 41](#_Toc107522109)

[References 42](#_Toc107522110)

# Table S1. Strains and plasmids used in this study.

| **Strain and Plasmid** | **Description** | **Reference** |
| --- | --- | --- |
| **Strains** |  |  |
| *S. globisporus* C-1027 | Wild type lidamycin producing strain | (Hu et al., 1988) |
| C-1027**/**pSET152 | *S. globisporus* C-1027 with the control vector pSET152, Am^r^ | This work |
| OELuxR1R2 | *S. globisporus* C-1027 with the expression vector pL-C26-LuxR1R2, Am^r^ | This work |
| OELuxR1 | *S. globisporus* C-1027 with the expression vector pL-C26-LuxR1, Am^r^ | This work |
| OELuxR2 | *S. globisporus* C-1027 with the expression vector pL-C26-LuxR2, Am^r^ | This work |
| OETetR | *S. globisporus* C-1027 with the expression vector pL-C26-TetR, Am^r^ | This work |
| OELacI | *S. globisporus* C-1027 with the expression vector pL-C26-LacI, Am^r^ | This work |
| glaEKO | Mutant of *S. globisporus* C-1027 with disruption of cluster 26 *glaE* gene (*SGL6306*), Ts^r^ | This work |
| echAKO | Mutant of *S. globisporus* C-1027 with disruption of cluster 26 *echA* gene (*SGL6301*), Ts^r^ | This work |
| orf3KO | Mutant of *S. globisporus* C-1027 with disruption of cluster 26 *orf3* gene (*SGL6292*), Ts^r^ | This work |
| *Escherichia coli* DH5α | General cloning host | TransGen |
| *E. coli* ET12567/pUZ8002 | Strain used for *E. coli*/*S. globisporus* conjugation | (Kieser et al., 2000) |
| **Plasmids** |  |  |
| pEASY-Blunt-Zero | General cloning vector, Amp^r^/Km^r^ | TransGen |
| pSET152 | *E. coli*/*Streptomyces* shuttle vector, Am^r^ | (Bierman et al., 1992) |
| pICLSet | pSET152 derivative containing *ermE**p and the ribosome-binding site of the *tuf1* gene up, Am^r^ | (Hong et al., 2007) |
| pL-C26-LuxR1R2 | pICLSet derivative containing coding region of *luxR1* and *luxR2* gene of cluster 26, Am^r^ | This work |
| pL-C26-LuxR1 | pICLSet derivative containing coding region of *luxR1* gene of cluster 26, Am^r^ | This work |
| pL-C26-LuxR2 | pICLSet derivative containing coding region of *luxR2* gene of cluster 26, Am^r^ | This work |
| pL-C26-TetR | pICLSet derivative containing coding region of *tetR* gene of cluster 26, Am^r^ | This work |
| pL-C26-LacI | pICLSet derivative containing coding region of *lacI* gene of cluster 26, Am^r^ | This work |
| pUC119 | General cloning vector, Amp^r^ | Previous work |
| pKC1139 | *E. coli/Streptomyces* shuttle vector with temperature-sensitive replication origin, Am^r^ | (Bierman et al., 1992) |
| pKC-LtR | pKC1139-derivative disrupting plasmid used for gene disruption of *glaE* gene, Am^r^, Ts^r^ | This work |
| pKC-AtB | pKC1139-derivative disrupting plasmid used for gene disruption of *echA* gene, Am^r^, Ts^r^ | This work |
| pKC-CtD | pKC1139-derivative disrupting plasmid used for gene disruption of *orf3* gene, Am^r^, Ts^r^ | This work |

*Abbreviations: Amp^r^, ampicillin resistance; Am^r^, apramycin resistance; Km^r^, kanamycin resistance; Ts^r^, thiostrepton resistance.

# Table S2. PCR primers used in this study.

| **Name** | **Sequence (5’-3’)** | **Purpose** |
| --- | --- | --- |
| **Plasmid construction** |  |  |
| Cluster26_luxR1R2_F | TAcatatgATGGCAGTGGACCAGCCT | Forward and reverse primers for *luxR1* and *luxR2* over expression in *S. globisporus*. |
| Cluster26_luxR1R2_R | ATggatccGGATTCAGCTGTCCGGGC |  |
| Cluster26_luxR1_F | TAcatatgGCAGTGGACCAGCCTGACCT | Forward and reverse primers for *luxR1* over expression in *S. globisporus*. |
| Cluster26_luxR1_R | ATggatccTCACGCGGGCAGCAGGCCGA |  |
| Cluster26_luxR2_F | TAcatatgCTGGACCGACGCGAGGACAA | Forward and reverse primers for *luxR2* over expression in *S. globisporus*. |
| Cluster26_luxR2_R | ATggatccTCAGCTGTCCGGGCGGCTGC |  |
| Cluster26_tetR_F | TAcatatgGTGCGCCGCCGCCTGCTGAA | Forward and reverse primers for *tetR* over expression in *S. globisporus*. |
| Cluster26_tetR_R | ATggatccGCCCCCTGCCGACGAGTTCC |  |
| Cluster26_lacI_F | TAcatatgATGGCGAGCATCAAAGATGT | Forward and reverse primers for *lacI* over expression in *S. globisporus*. |
| Cluster26_lacI_R | GAtctagaAGGTCGAAGTCGACGTTGTC |  |
| Cluster26_glaEKO_LF | CGaagcttACATGTAGACCGGCGGATAG | Used for amplifying 1685 bp upstream arm used in knockout of *glaE* gene |
| Cluster26_glaEKO_LR | GAtctagaGAGCCCGGTTATGACTACTC |  |
| Cluster26_glaEKO_RF | GAtctagaGTGATCTTCGGCAAGTACG | Used for amplifying 1537 bp downstream arm used in knockout of *glaE* gene |
| Cluster26_glaEKO_RR | TAggatccGGTCTCCTTGATCCCGTCC |  |
| Cluster26_echAKO_LF | CGaagcttGACCTGTACGAGTCCTTCCC | Used for amplifying 1797 bp upstream arm used in knockout of *echA* |
| Cluster26_echAKO_LR | GAtctagaAACGCGAAGAAGATCCTCGT |  |
| Cluster26_echAKO_RF | GAtctagaCCTGGTCAACAAATCCGCAT | Used for amplifying 1557 bp downstream arm used in knockout of *echA* |
| Cluster26_echAKO_RR | TAggatccCCGGTTCGAGAAATTCCACG |  |
| Cluster26_orf3KO_LF | CGaagcttATACGTAGCGGTGTGATGGG | Used for amplifying 1568 bp upstream arm used in knockout of *orf3 gene* |
| Cluster26_orf3KO_LR | GAtctagaGAGCCGCCAGTACTGATTCA |  |
| Cluster26_orf3KO_RF | GAtctagaGCCGACGCGATATTCCATC | Used for amplifying 2095 bp downstream arm used in knockout of *orf3 gene* |
| Cluster26_orf3KO_RR | TAggatccCGCAGCGACACATTGATCT |  |
| **RT-qPCR** |  |  |
| hrdB_RT | TGGTCGAGGTCATCAACAAG | Used for the detection of *hrdB* transcripts |
| hrdB_RT | TGGACCTCGATGACCTTCTC |  |
| cluster26_orf1_RTF | CTGCTGCTCTTCGTCGTCTA | Used for amplifying 105 bp coding region of *orf1* |
| cluster26_orf1_RTR | CATCCTGCCGAGAACCCG |  |
| cluster26_orf2_RTF | CGATGTGACACGCCTGGT | Used for amplifying 148 bp coding region of *orf2* |
| cluster26_orf2_RTR | GTCCAGGAGCCACAGGTAG |  |
| cluster26_orf3_RTF | GGACACGATGGCACTTCTG | Used for amplifying 111 bp coding region of *orf3* |
| cluster26_orf3_RTR | AGATCGATGAGCAGCCGG |  |
| cluster26_luxR1_RTF | AGCCTGACCTGAACGTGG | Used for amplifying 101 bp coding region of *luxR1* |
| cluster26_luxR1_RTR | GACGGTGGACTCGATCAGA |  |
| cluster26_luxR2_RTF | GAGGACAAACTCGAACTGGC | Used for amplifying 106 bp coding region of *luxR2* |
| cluster26_luxR2_RTR | CCACCGATGCGATCAGTTG |  |
| cluster26_echA_RTF | GAAGCGACACGGAACATCTG | Used for amplifying 182 bp coding region of *echA* |
| cluster26_echA_RTR | GAGGCTGGTCTTGGGGAAG |  |
| cluster26_echB_RTF | CAGTTGAACGTCCTGGGGA | Used for amplifying 163 bp coding region of *echB* |
| cluster26_echB_RTR | CGAGCGAGTTCACTTCGATG |  |
| cluster26_glaB_RTF | GAAAGCCGCTCCATCAGG | Used for amplifying 153 bp coding region of *glaB* |
| cluster26_glaB_RTR | GAGTCAGCAGGCTTTCGC |  |
| cluster26_glaC_RTF | CTGACCGACGCCATCCAC | Used for amplifying 128 bp coding region of *glaC* |
| cluster26_glaC_RTR | TCGTAGAAGGTCCGCTCCA |  |
| cluster26_glaD_RTF | ACGACGTCACCCAGGAAAG | Used for amplifying 106 bp coding region of *glaD* |
| cluster26_glaD_RTR | TTGATGACCACGTTGAAGGC |  |
| cluster26_glaE_RTF | AGTGGATCCGCAACTTCGA | Used for amplifying 141 bp coding region of *glaE* |
| cluster26_glaE_RTR | TGGTGCCGATGGAGATCAG |  |
| cluster26_glaQ_RTF | CTGCTCCAGGACTCCGAG | Used for amplifying 109 bp coding region of *glaQ* |
| cluster26_glaQ_RTR | CACGGGTGAAGTCCATGATG |  |
| cluster26_glaU_RTF | TGAAGGACGAGGACTGGAAC | Used for amplifying 118 bp coding region of *glaU* |
| cluster26_glaU_RTR | GAGATGTTCACGATGCAGCC |  |
| cluster26_glaW_RTF | GAACGCGTCCTGACCCAC | Used for amplifying 100 bp coding region of *glaW* |
| cluster26_glaW_RTR | GCAGGTAGAACGACGGGAA |  |
| cluster26_glaZ_RTF | CGGCAGATGTTCACGATCAT | Used for amplifying 111 bp coding region of *glaZ* |
| cluster26_glaZ_RTR | CACCCTGGACTTGGCGTA |  |
| cluster26_orf9_RTF | TCCGTACAGCTGGAGATCC | Used for amplifying 150 bp coding region of *orf9* |
| cluster26_orf9_RTR | GTGCTCGTTGACCACCAG |  |
| cluster26_orf11_RTF | GACATGCTGCTCTTCTTCCG | Used for amplifying 102 bp coding region of *orf11* |
| cluster26_orf11_RTR | GCCCGGCTCGATGTACTC |  |
| cluster26_tetR_RTF | CGAACTCTGCGGTGTCTAC | Used for amplifying 103 bp coding region of *tetR* |
| cluster26_tetR_RTR | CTCGTCGGTGAGTGTGAAGC |  |
| cluster26_lacI_RTF | CCCTCGGCCTGGTCATCA | Used for amplifying 106 bp coding region of *lacI* |
| cluster26_lacI_RTR | TTGCCGATGATGACGCTGTA |  |
|  |  |  |
| **PCR verification** |  |  |
| pSET152 | TTCGGCGGCTTCAAGTTCGG | Used for amplifying 1.6 kb pSET152-attB integration site |
| AttB-Strep | CGGTGGGGGTGCCAGGG |  |
| glaE_F1 | AGTAGTCATAACCGGGCTC | Used for amplifying 1572 bp of glaEKO |
| glaE_R1 | TTGGAGAAGGTGTAGGTCCG |  |
| glaE_LP1 | AGTGCGTACGGGCTGAAC | Used for amplifying 1997 bp of glaEKO |
| glaE_LP2 | GACGGATTTGCGATGGTGTC |  |
| glaE_RP1 | AGGATCGACAGGAATCTCGC | Used for amplifying 1830 bp of glaEKO |
| glaE_RP2 | GACACCGATCACCACGAAC |  |
| echA_F1 | AGACACCAGACTTCGTTCCC | Used for amplifying 1365 bp of echAKO |
| echA_R1 | GACCTACTGTTCTCCCCGAG |  |
| echA_LP1 | CTACCGTTCCCACCGCAC | Used for amplifying 1988 bp of echAKO |
| echA_LP2 | ATGATGAGCCAGACCGAGTC |  |
| echA_RP1 | TCGTCGCTCTCTGGCAAA | Used for amplifying 1777 bp of echAKO |
| echA_RP2 | GTCTTCATGCGGCAGTGG |  |
| orf3_F1 | CCGCCATGATCCAGGACTT | Used for amplifying 1935 bp of orf3KO |
| orf3_R1 | CATCATCCTGCACGAACTGG |  |
| orf3_LP1 | GCGAGAGGTTGAAGCGGT | Used for amplifying 1793 bp of orf3KO |
| orf3_LP2 | ATGATGAGCCAGACCGAGTC |  |
| orf3_RP1 | GACGGATTTGCGATGGTGTC | Used for amplifying 2355 bp of orf3KO |
| orf3_RP2 | GCAGTATCTCCGCGACGTAT |  |

^a)^ The restriction sites introduced by these oligonucleotides are underlined.

# Table S3. Predicted Secondary metabolite biosynthetic gene clusters in *S. globisporus* C-1027 by antiSMASH.

| **Cluster** | **type** | **start** | **end** | **Most similar known cluster** | **similarity** |
| --- | --- | --- | --- | --- | --- |
| Cluster 1 | Terpene | 103527 | 125344 | 2-methylisoborneol | 75 % |
| Cluster 2 | Butyrolactone | 177217 | 188161 | gamma-butyrolactone | 100 % |
| Cluster 3 | Terpene | 222188 | 244401 | Stenothricin | 13 % |
| Cluster 4 | Siderophore | 284629 | 341455 | Griseobactin | 100 % |
| Cluster 5 | Siderophore | 333122 | 384170 | Coelichelin | 81 % |
| Cluster 6 | Terpene | 383711 | 409903 | Isorenieratene | 100 % |
| Cluster 7 | NRPS | 400994 | 451409 | Griseoviridin | 15 % |
| Cluster 8 | t3PKS | 472394 | 513512 | Herboxidiene | 6 % |
| Cluster 9 | Terpene | 853859 | 879834 | Isorenieratene | 85 % |
| Cluster 10 | NRPS | 871087 | 917470 | Tetronasin | 3 % |
| Cluster 11 | Terpene | 1181387 | 1202688 | Steffimycin | 19 % |
| Cluster 12 | Ectoine | 1631052 | 1641480 | Ectoine | 100 % |
| Cluster 13 | Lantipeptide | 2652753 | 2675896 | - | - |
| Cluster 14 | Siderophore | 2742719 | 2754497 | Desferrioxamine B | 80 % |
| Cluster 15 | Lantipeptide | 3122859 | 3154609 | - | - |
| Cluster 16 | NRPS | 3335176 | 3400737 | Oxazolomycin | 6 % |
| Cluster 17 | Lassopeptide | 4463964 | 4486667 | SRO15-2005 | 60 % |
| Cluster 18 | Lantipeptide | 4552520 | 4594559 | A54145 | 3 % |
| Cluster 19 | NRPS-t1PKS | 5167813 | 5222510 | Himastatin | 12 % |
| Cluster 20 | Lantipeptide | 5299614 | 5322418 | AmfS | 100 % |
| Cluster 21 | Ectoine | 5532667 | 5543041 | Pristinamycin | 23 % |
| Cluster 22 | Terpene | 5664830 | 5686107 | - | - |
| Cluster 23 | Siderophore | 6082612 | 6097344 | - | - |
| Cluster 24 | Bacteriocin | 6331423 | 6342799 | - | - |
| Cluster 25 | Ectoine | 6564114 | 6574599 | Kosinostatin | 11 % |
| Cluster 26 | NRPS-PKS | 6765985 | 6819639 | Skyllamycin | 24 % |
| Cluster 27 | Terpene | 6959815 | 6986388 | Hopene | 69 % |
| Cluster 28 | NRPS-t1PKS | 7040610 | 7090005 | SGR_PTMs | 83 % |
| Cluster 29 | Bacteriocin | 7089389 | 7100936 | - | - |
| Cluster 30 | Melanin | 7287746 | 7298213 | Melanin | 100 % |
| Cluster 31 | t3PKS | 7337365 | 7378504 | Tetronasin | 11 % |
| Cluster 32 | NRPS-t1PKS | 7483759 | 7536094 | Daptomycin | 7 % |
| Cluster 33 | NRPS-t1PKS | P1-8213 | P1-104675 | Lidamycin | 100 % |

# Table S4. Annotation and predicted function of genes in cluster 26.

| **Gene** | **Name** | **Size (aa)** | **Proposed Function** | **BLAST Closest Homolog (identity %)** | **other BGC’s proteins (identity %)** |
| --- | --- | --- | --- | --- | --- |
| SGL6289 | Orf 1 | 434 | Unknown | WP_097877802.1 (*Streptomyces* sp. ms184) (99 %) |  |
| SGL6290 | Orf 2 | 263 | Unknown | WP_083193622.1 (*Streptomyces violaceoruber*) (95 %) |  |
| SGL6291 | TetR | 208 | TetR family transcriptional regulator | SDC74001.1 (*Streptomyces* sp. LaPpAH-199) (97 %) |  |
| SGL6292 | Orf 3 | 1161 | Beta-ketoacyl synthase (KS) | WP_073822031.1 (*Streptomyces* sp. CB02130) (99 %) |  |
| SGL6293 | Orf 4 | 184 | Unknown | WP_083193624.1 (*Streptomyces violaceoruber*) (95 %) |  |
| SGL6294 | Orf 5 | 146 | Unknown | WP_098008453.1 (*Streptomyces* sp. sk226) (96 %) |  |
| SGL6295 | LuxR1 | 236 | LuxR family transcriptional regulator | KOU03118.1 (*Streptomyces* sp. NRRL F-2295) (94 %) | Skyllamycin BGC: Sky47 (42 %); Atratumycin BGC: Atr1 (47 %); Cinnapeptin BGC: Cip2 (46 %) |
| SGL6296 | LuxR2 | 267 | LuxR family transcriptional regulator | WP_073822030.1 (*Streptomyces* sp. CB02130) (99 %) | Skyllamycin BGC: Sky46 (48 %); Atratumycin BGC: Atr2 (47 %); Cinnapeptin BGC: Cip3 (44 %) |
| SGL6297 | Orf 6 | 388 | Unknown | WP_073822029.1 (*Streptomyces* sp. CB02130) (99 %) |  |
| SGL6300 | EchC | 134 | SnoaL-like domain-containing protein | WP_050497604.1 (*Streptomyces* *griseus*) (90 %) | Echosides BGC: EchC (66 %) |
| SGL6299 | EchB | 308 | NAD-dependent epimerase/dehydratase | WP_032791797.1 (*Streptomyces griseus*) (97 %) | Echosides BGC: EchB (77 %) |
| SGL6301 | EchA | 919 | Peptide synthetase (NRPS, A-T-TE) | WP_073822028.1 (*Streptomyces* sp. CB02130) (99 %) | Echosides BGC: EchA(66 %) |
| SGL6302 | GlaA | 312 | Malonyl-CoA ACP transferase (AT) | WP_073822027.1 (*Streptomyces* sp. CB02130) (99 %) | Skyllamycin BGC: Sky13 (61 %); Atratumycin BGC: Atr3 (67 %); Cinnapeptin BGC: Cip4 (64 %);  Youssoufenes BGC: YsfN (39 %) |
| SGL6303 | GlaB | 283 | Phosphopantetheinyl transferase (PPTase) | SDC73663.1 (*Streptomyces* sp. LaPpAH-199) (90 %) | Cinnapeptin BGC: Cip1 (35 %) |
| SGL6304 | GlaC | 481 | Cytochrome P450 | WP_073822026.1 (*Streptomyces* sp. CB02130) (99 %) |  |
| SGL6305 | GlaD | 84 | Acyl carrier protein (ACP) | WP_097900294.1 (*Streptomyces* sp. b94) (99 %) | Skyllamycin BGC: Sky16 (72 %); Youssoufenes BGC: YsfA (55 %); Atratumycin BGC: Atr5 (77 %); Cinnapeptin BGC: Cip6 (77 %) |
| SGL6306 | GlaE | 410 | Beta-ketoacyl synthase (KS) | WP_030190764.1 (*Streptomyces griseus*) (99 %) | Skyllamycin BGC: Sky17 (74 %); Youssoufenes BGC: YsfB (52 %); Atratumycin BGC: Atr6 (73 %); Cinnapeptin BGC: Cip7 (74 %) |
| SGL6307 | GlaF | 396 | Beta-ketoacyl synthase (KS) | SDC73504.1 (*Streptomyces* sp. LaPpAH-199) (96 %) | Skyllamycin BGC: Sky18 (58 %); Youssoufenes BGC: YsfD (42 %); Atratumycin BGC: Atr7 (67 %); Cinnapeptin BGC: Cip8 (66 %) |
| SGL6308 | GlaG | 315 | Chain length factor (CLF) | WP_073822025.1 (*Streptomyces* sp. CB02130) (99 %) | Skyllamycin BGC: Sky19 (51 %); Youssoufenes BGC: YsfE (49 %); Atratumycin BGC: Atr8 (53 %); Cinnapeptin BGC: Cip9 (54 %) |
| SGL6309 | GlaH | 286 | α/β hydrolase | WP_073822024.1 (*Streptomyces* sp. CB02130) (99 %) | Skyllamycin BGC: Sky20 (45 %); Atratumycin BGC: Atr9 (49 %); Cinnapeptin BGC: Cip10 49 %) |
| SGL6310 | GlaI | 336 | Unknown | WP_030846637.1 (*Streptomyces griseus*) (97 %) | Skyllamycin BGC: Sky 21 (47 %); Youssoufenes BGC: YsfF (41 %); Atratumycin BGC: Atr10 (63 %); Cinnapeptin BGC: Cip11 (64 %) |
| SGL6311 | GlaJ | 395 | Beta-ketoacyl synthase (KS) | WP_043248289.1 (*Streptomyces vinaceus*) (98 %) | Youssoufenes BGC: YsfJ (45 %) |
| SGL6312 | GlaK | 335 | Chain length factor (CLF) | WP_098008462.1 (*Streptomyces* sp. sk226) (97 %) | Youssoufenes BGC: YsfK (39 %) |
| SGL6313 | GlaL | 264 | short-chain dehydrogenase/reductase (SDR) | WP_073822023.1 (*Streptomyces sp.* CB02130) (99 %) |  |
| SGL6314 | GlaM | 332 | α/β hydrolase | WP_073822022.1 (*Streptomyces sp.* CB02130) (99 %) |  |
| SGL6315 | GlaN | 108 | Unknown | WP_073822021.1 (*Streptomyces sp.* CB02130) (99 %) | Cinnapeptin BGC: Cip12 (43 %) |
| SGL6316 | GlaO | 126 | Unknown | WP_030734252.1 (*Streptomyces griseus*) (99 %) | Cinnapeptin BGC: Cip13 (50 %) |
| SGL6317 | GlaP | 134 | Unknown | WP_093901642.1 (*Streptomyces sp*. LaPpAH-199) (97 %) | Cinnapeptin BGC: Cip14 (54 %) |
| SGL6318 | GlaQ | 363 | Chain length factor (CLF) | WP_073822020.1 (*Streptomyces sp*. CB02130) (99 %) | Skyllamycin BGC: Sky 22 (56 %); Youssoufenes BGC: YsfC (38 %); Atratumycin BGC: Atr11 (55 %); Cinnapeptin BGC: Cip15 (57 %) |
| SGL6319 | GlaR | 86 | Acyl carrier protein (ACP) | WP_030590804.1 (*Streptomyces anulatus*) (88 %) | Skyllamycin BGC: Sky 23 (52 %); Youssoufenes BGC: YsfA (52 %); Atratumycin BGC: Atr12 (68 %); Cinnapeptin BGC: Cip16 (67 %) |
| SGL6320 | GlaS | 127 | Dehydratase (DH) | WP_030292460.1 (*Streptomyces* sp. NRRL F-5681) (94 %) | Skyllamycin BGC: Sky24 (47 %); Atratumycin BGC: Atr13 (56 %); Cinnapeptin BGC: Cip17 (53 %) |
| SGL6321 | GlaT | 168 | Dehydratase (DH) | WP_073822220.1 (*Streptomyces* sp. CB02130) (99 %) | Skyllamycin BGC: Sky 25 (53 %); Youssoufenes BGC: YsfI (35 %); Atratumycin BGC: Atr14 (63 %); Cinnapeptin BGC: Cip18 (64 %) |
| SGL6322 | GlaU | 239 | Ketoreductase (KR) | WP_098008467.1 (*Streptomyces* sp. sk226) (98 %) | Skyllamycin BGC: Sky 26 (65 %); Youssoufenes BGC: YsfH (60 %); Atratumycin BGC: Atr15 (65 %); Cinnapeptin BGC: Cip19 (65 %) |
| SGL6323 | GlaV | 233 | Isomerase | SDC73017.1 (*Streptomyces* sp. LaPpAH-199) (94 %) | Skyllamycin BGC: Sky 27 (57 %); Youssoufenes BGC: YsfG (47 %); Atratumycin BGC: Atr16 (62 %); Cinnapeptin BGC: Cip20 (60 %) |
| SGL6324 | GlaW | 293 | α/β hydrolase | WP_030292453.1 (*Streptomyces* sp. NRRL F-5681) (97 %) | Cinnapeptin BGC: Cip5 (42 %) |
| SGL6325 | GlaX | 181 | Unknown | WP_098008470.1 (*Streptomyces* sp. sk226) (96 %) |  |
| SGL6326 | GlaY | 516 | Major Facilitator Superfamily transporter | WP_083193635.1 (*Streptomyces violaceoruber*) (98 %) |  |
| SGL6327 | GlaZ | 608 | Acyl-CoA dehydrogenase | WP_032772334.1 (*Streptomyces* sp. NRRL F-2202) (98 %) |  |
| SGL6328 | LacI | 338 | LacI family transcriptional regulator | WP_097876986.1 (*Streptomyces* sp. ms184) (99 %) |  |
| SGL6329 | Orf 7 | 522 | ABC transporter ATP-binding protein | WP_086672578.1 (*Streptomyces albovinaceus*) (99 %) |  |
| SGL6330 | Orf 8 | 635 | Transporter | WP_097876989.1 (*Streptomyces* sp. ms184) (99 %) |  |
| SGL6331 | Orf 9 | 307 | PfkB family carbohydrate kinase | WP_097876991.1 (*Streptomyces* sp. ms184) (99 %) |  |
| SGL6332 | Orf 10 | 129 | Unknown | WP_097985125.1 (*Streptomyces* sp. f150) (98 %) |  |
| SGL6333 | Orf 11 | 208 | Unknown | WP_097973898.1 (*Streptomyces* sp. gb14) (98 %) |  |

# Table S5. ^1^H and ^13^C NMR data for compounds 1-3 in (CD_3_)_2_CO.

|  | **compound 1** | | **compound 2** | | **compound 3** | |
| --- | --- | --- | --- | --- | --- | --- |
| **Pos.** | ***δ*_H_, multi (*J* in Hz)** | ***δ*_C_, type** | ***δ*_H_ multi (*J* in Hz)** | ***δ*_C_, type** | ***δ*_H_ multi (*J* in Hz)** | ***δ*_C_, type** |
| **1** |  | 174.9, C |  | 173.8, C |  | 167.5, C |
| **2** | 2.28, t (7.0) | 34.4, CH_2_ | 2.53, m | 34.0, CH_2_ | 6.41, d (13.5) | 120.1, CH |
| **3** | 1.59, m | 25.8, CH_2_ | 2.51, m | 29.1, CH_2_ | 7.96, d (13.5) | 142.8, CH |
| **4** | 1.29-1.39, m | 30.4 ^a^, CH_2_ | 6.12, dt (13.5, 5.5) | 131.0, CH | - | 134.1, C |
| **5** | 1.29-1.39, m | 30.0 ^a^, CH_2_ | 6.70, dt (13.5, 1.5) | 130.8, CH | - | 138.2, C |
| **6** | 1.29-1.39, m | 30.0 ^a^, CH_2_ | - | 137.4, C | 7.30, d (7.5) | 127.1, CH |
| **7** | 1.29-1.39, m | 30.2 ^a^, CH_2_ | - | 135.5, C | 7.27, m | 130.7, CH |
| **8** | 1.29-1.39, m | 30.3 ^a^, CH_2_ | 7.12, m | 127.7, CH | 7.27, m | 127.2, CH |
| **9** | 1.29-1.39, m | 30.4 ^a^, CH_2_ | 7.12, m | 129.2, CH | 7.69, d (7.5) | 131.5, CH |
| **10** | 1.50, m | 30.3, CH_2_ | 7.12, m | 126.7, CH | 2.45, s | 19.6, CH_3_ |
| **11** | 2.25, ddd (13.5, 5.5, 1.5) | 34.1, CH_2_ | 7.41, d (7.5) | 126.1, CH |  |  |
| **12** | 6.12, dt (13.5, 5.5) | 133.1, CH | 2.31, s | 19.7, CH_3_ |  |  |
| **13** | 6.62, dt (13.5, 1.5) | 131.0, CH |  |  |  |  |
| **14** | - | 137.9, C |  |  |  |  |
| **15** | - | 135.6, C |  |  |  |  |
| **16** | 7.02, m | 127.7, CH |  |  |  |  |
| **17** | 7.02, m | 128.7, CH |  |  |  |  |
| **18** | 7.02, m | 127.0 CH |  |  |  |  |
| **19** | 7.41, d (7.5) | 126.3, CH |  |  |  |  |
| **20** | 2.29, s | 20.0, CH_3_ |  |  |  |  |

^1^H and ^13^C NMR spectral data (*δ*) were obtained at 500 and 125 MHz, respectively. Proton coupling constants (*J*) in Hz were given in parentheses. The assignments were based on gCOSY, HSQC, and HMBC spectra. ^a^ These signals cannot be undoubtedly assigned to the specific carbons due to the severely overlapped signals of corresponding protons, however they can be incontrovertibly confirmed to be the methine carbon signals at positions C4−C9 based on their nearly identical chemical shifts and by comparison with the reported fatty acid carbon chemical shifts ^(Gunstone et al., 1977)^.


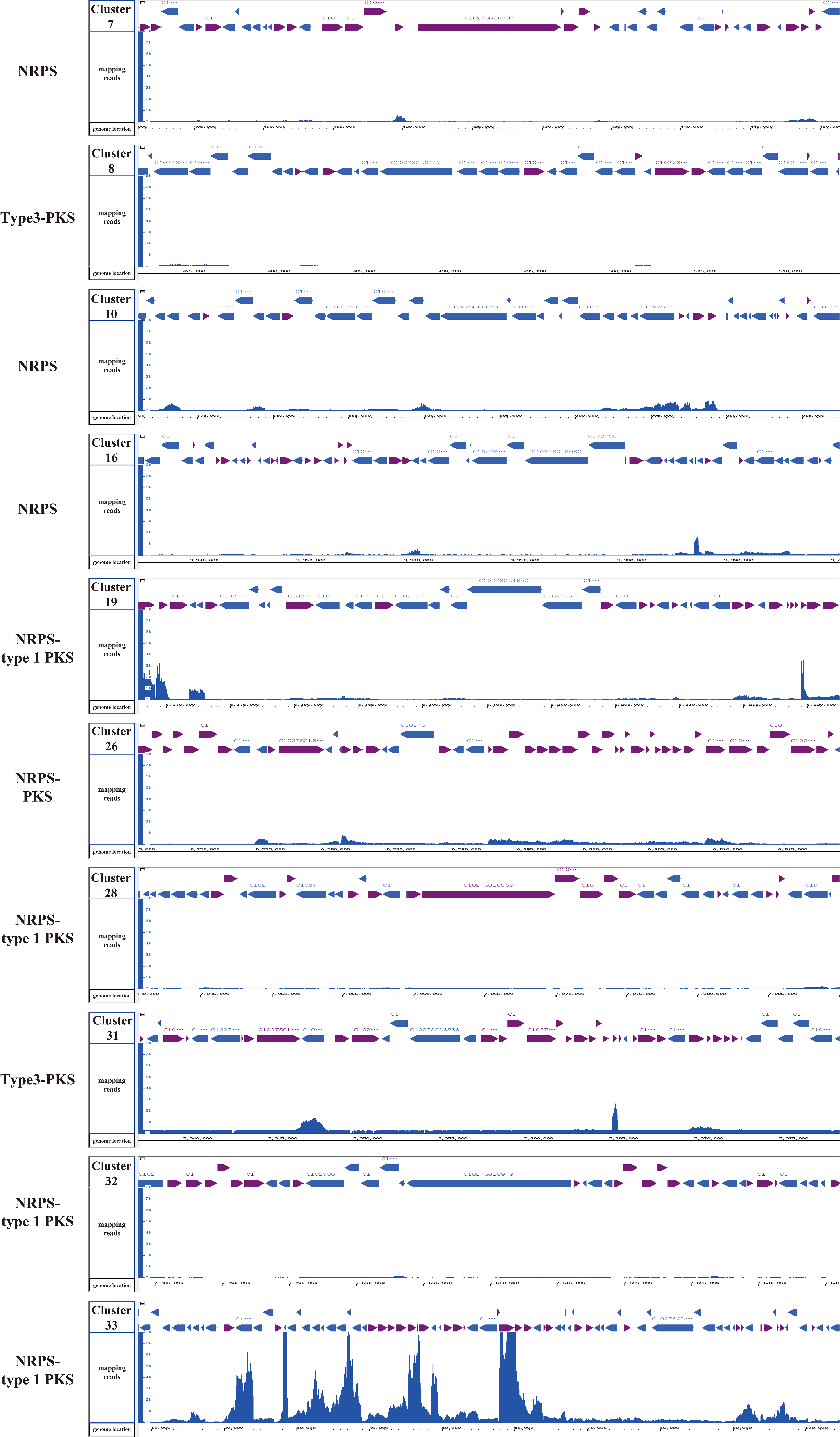


# Figure S1. Transcriptional analysis of secondary metabolites biosynthetic gene clusters in *S. globisporus* C-1027.

Ten NRPS/PKS biosynthetic gene clusters in *S. globisporus* C-1027 were predicted by antiSMASH and their expression level was analyzed by transcriptome sequencing. Strains were collected for the extraction of total RNAs at 48 h at the beginning of the fermentation in FMC-1027-1 medium. Illumina sequencing reads were aligned to the *S. globisporus* C-1027 reference genome. The distribution of the transcriptome reads was shown in the Integrated Genome Browser.

**
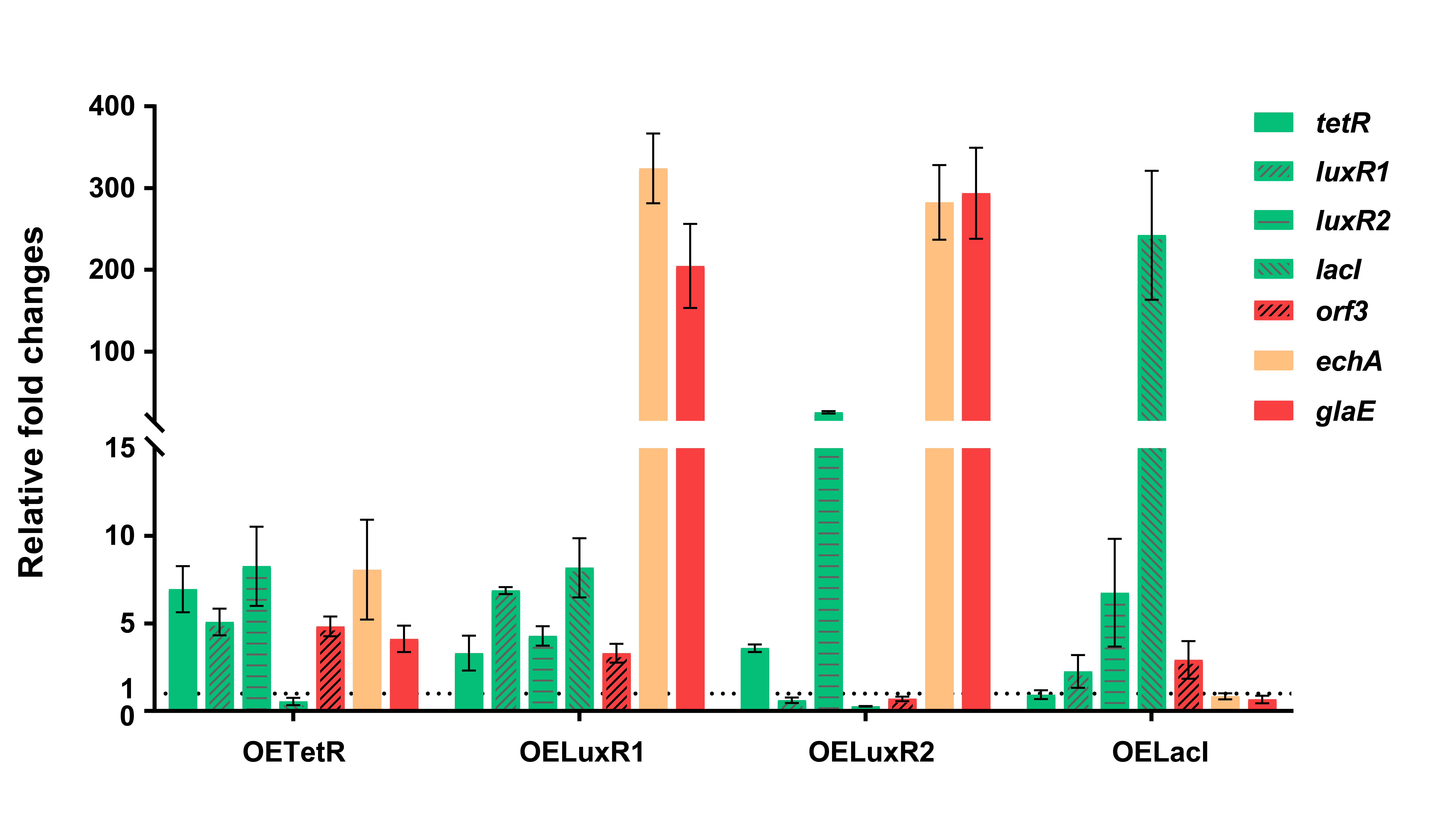
**

# Figure S2. Expression levels of cluster 26 genes in regulator-overexpression strains detected by RT-qPCR analysis.

The mycelia of four regulator-overexpression strains (OETetR, OELuxR1, OELuxR2, OELacI) were collected for the extraction of total RNAs at 48 h of the fermentation in FMC-1027-1 medium. These samples were subjected to RT-qPCR analysis. The relative mRNA level of the target genes was normalized to the principal sigma factor gene *hrdB*. The relative expression level of each sample was represented as the value related to the control strain C-1027/pSET152. Values were presented as means ± SEM (three biological repeats for each strain).

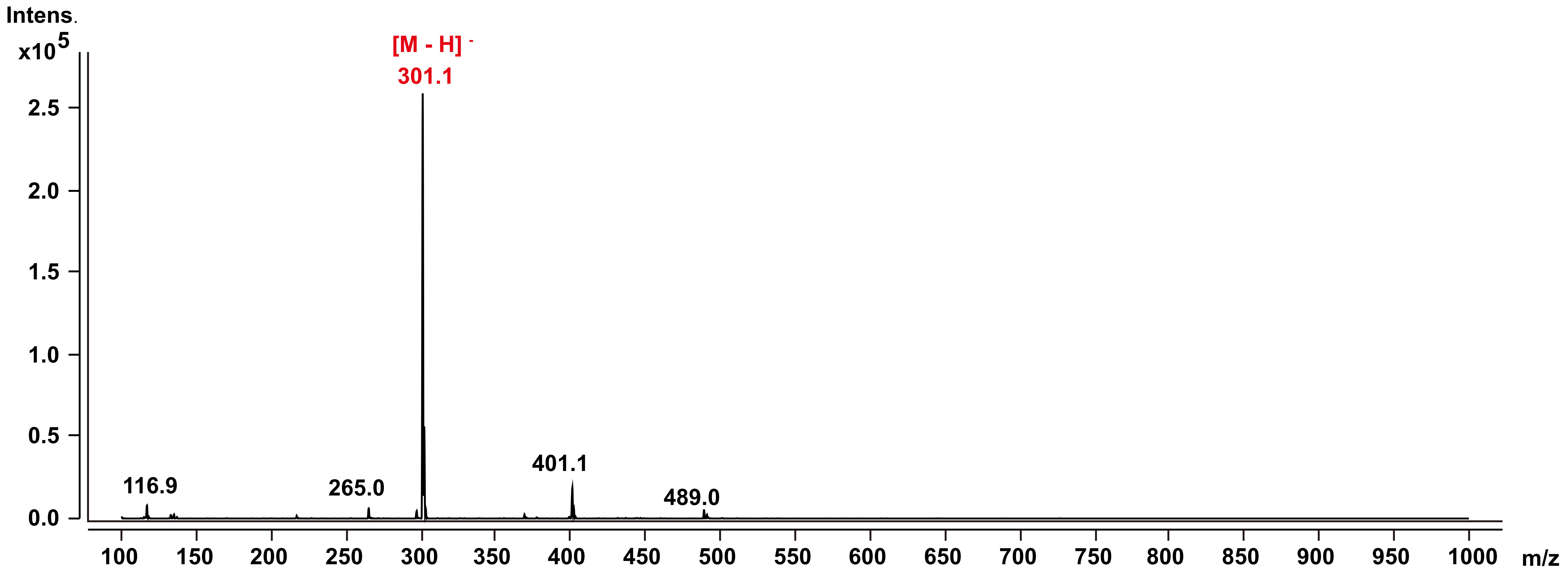


Figure S3. (-)-ESI-MS of compound 1.


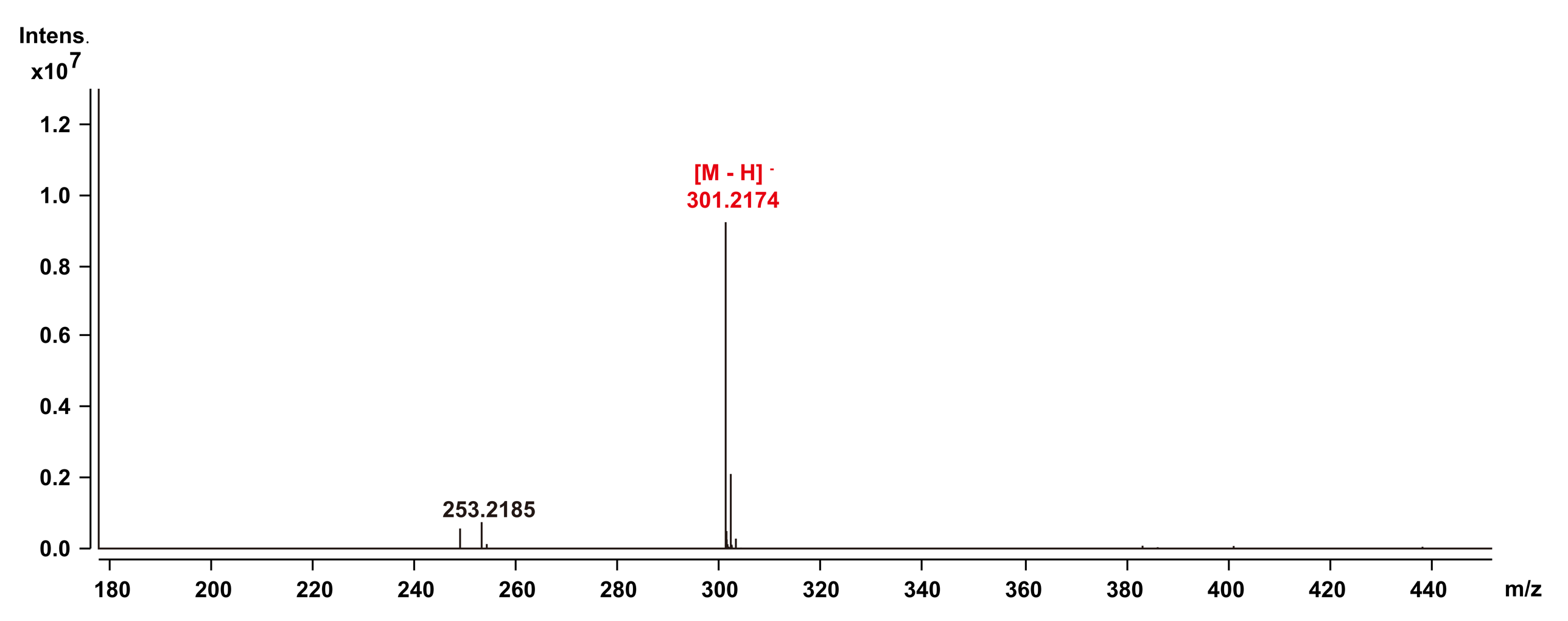


Figure S4. (-)-ESI-HRMS analysis of compound 1.

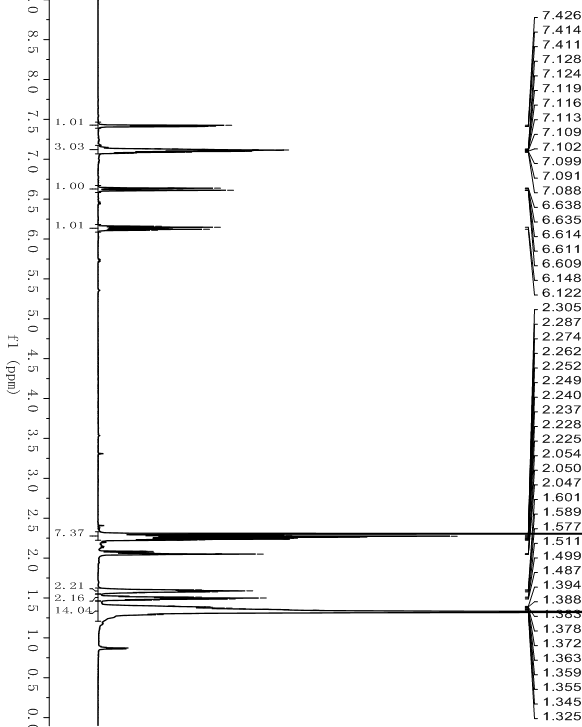


Figure S5. ^1^H-NMR spectrum of compound 1 (500 MHz, (CD_3_)_2_CO).

Figure S6. ^13^C-NMR spectrum of compound 1 (125 MHz, (CD_3_)_2_CO).

Figure S7. DEPT spectrum of compound 1 (125 MHz, (CD_3_)_2_CO).

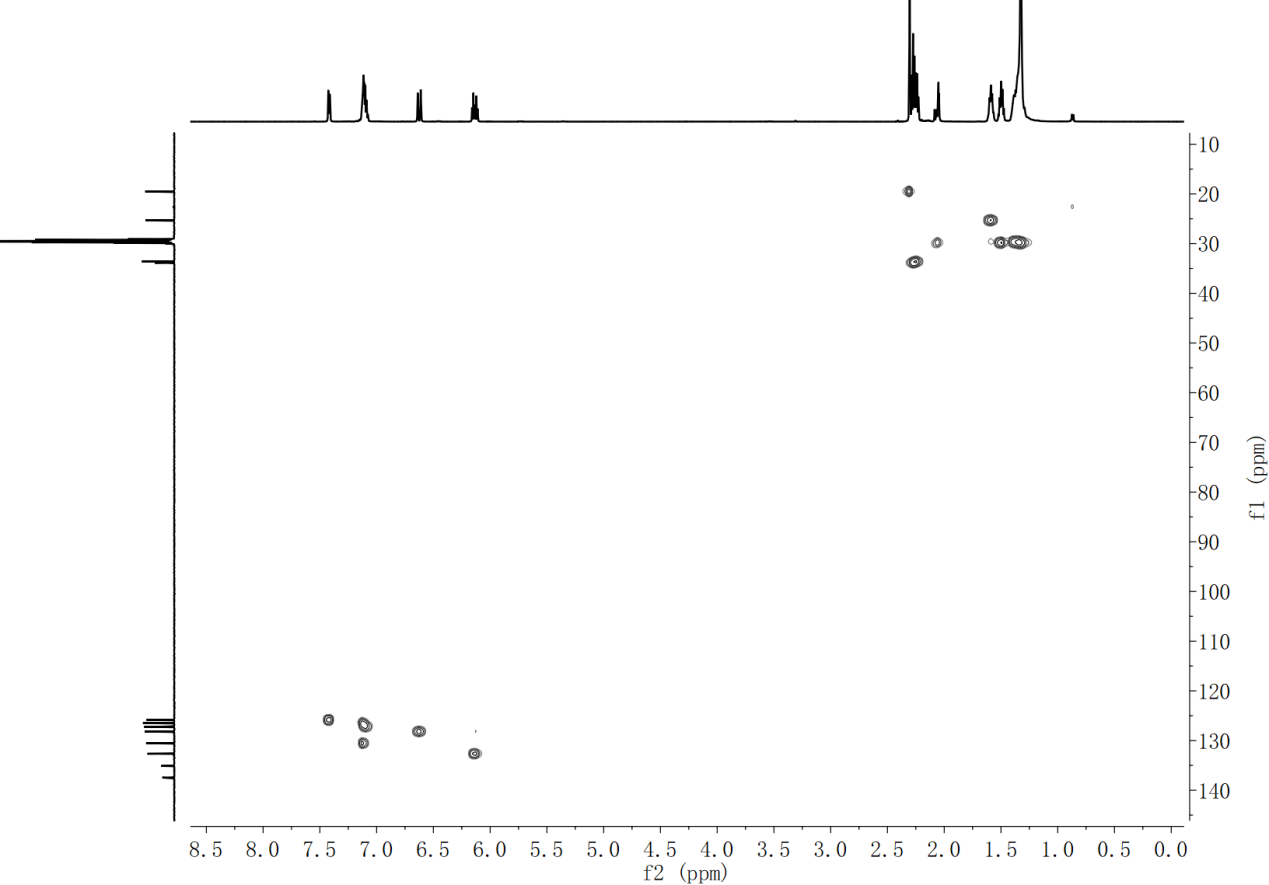


Figure S8. HSQC spectrum of compound 1 ((CD_3_)_2_CO).

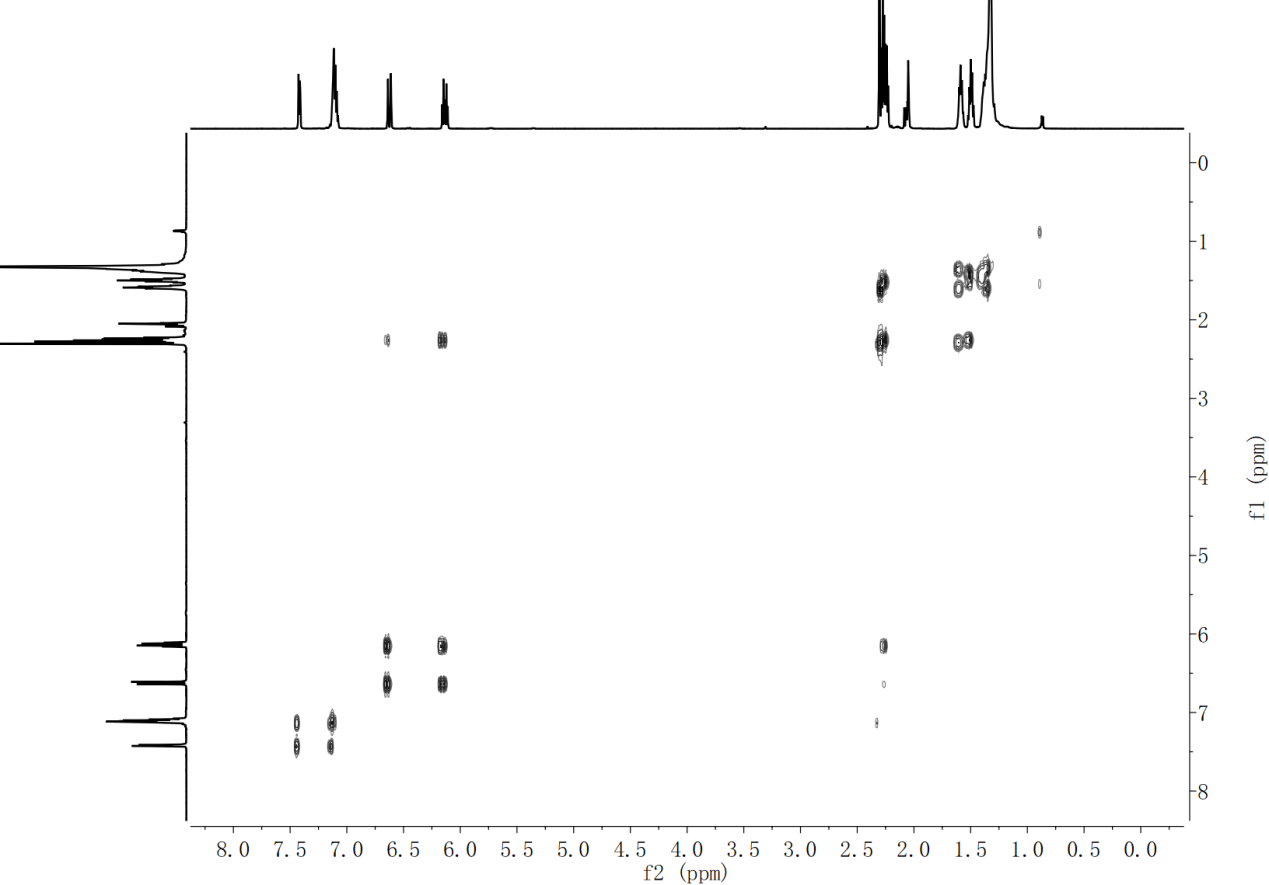
Figure S9. ^1^H-^1^H COSY spectrum of compound 1 ((CD_3_)_2_CO).

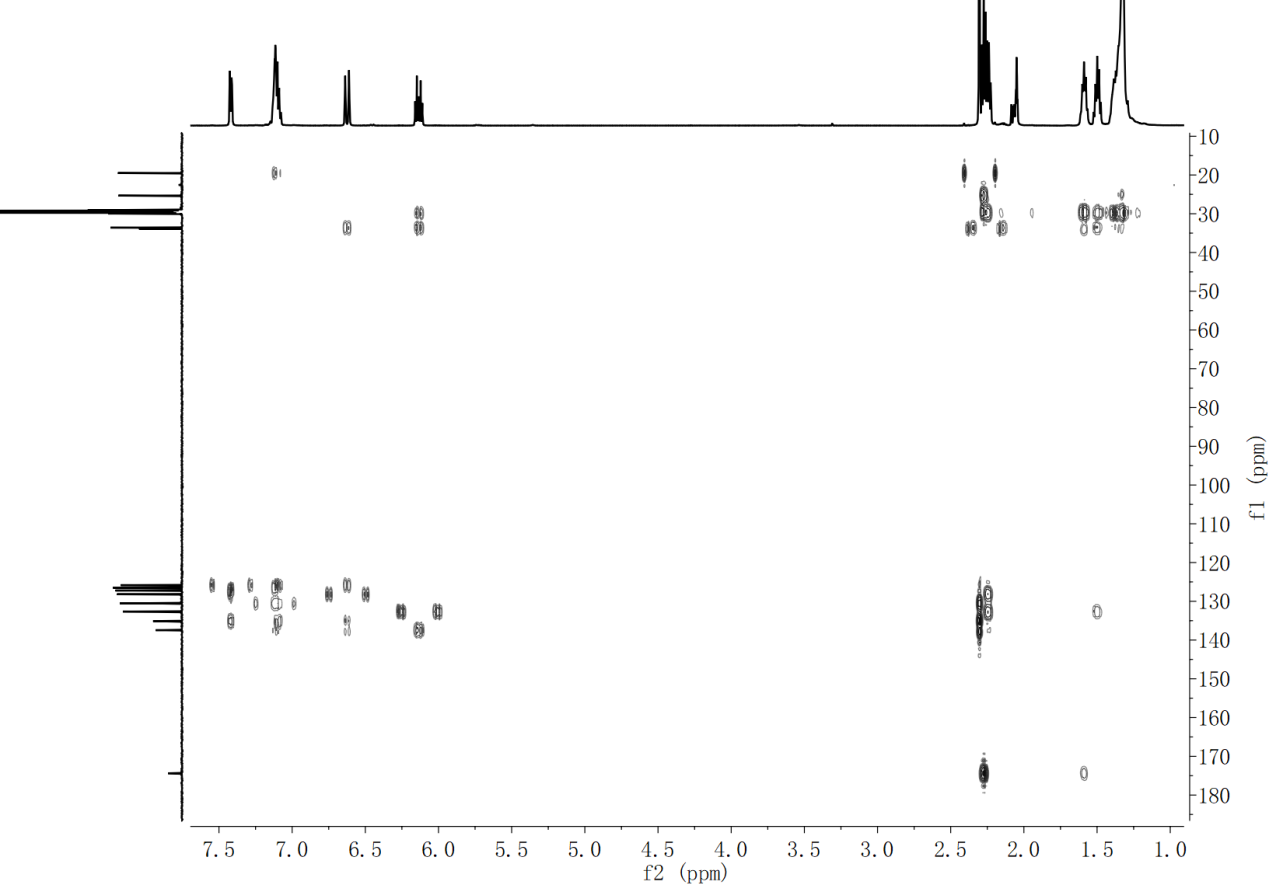
Figure S10. HMBC spectrum of compound 1 ((CD_3_)_2_CO).

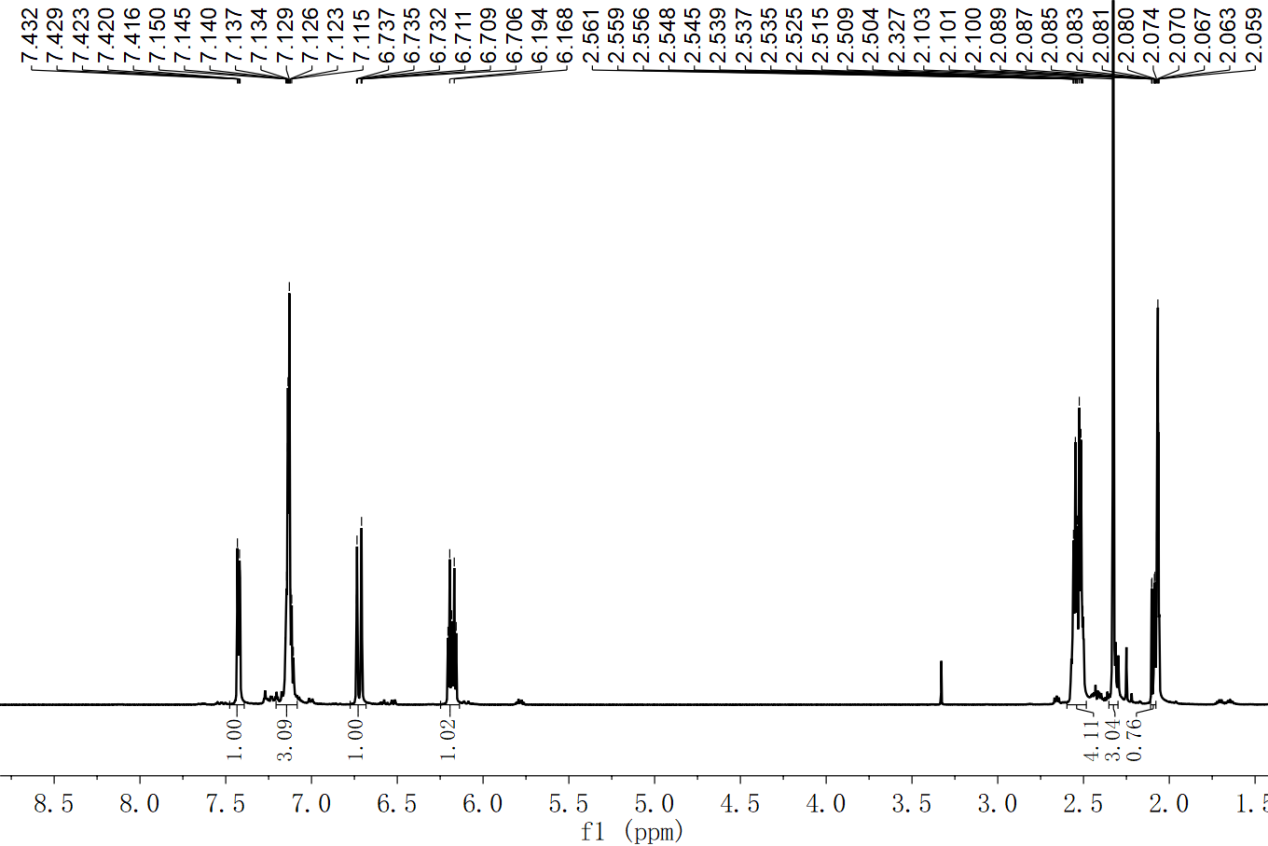


Figure S11. ^1^H-NMR spectrum of compound 2 (500 MHz, (CD_3_)_2_CO).

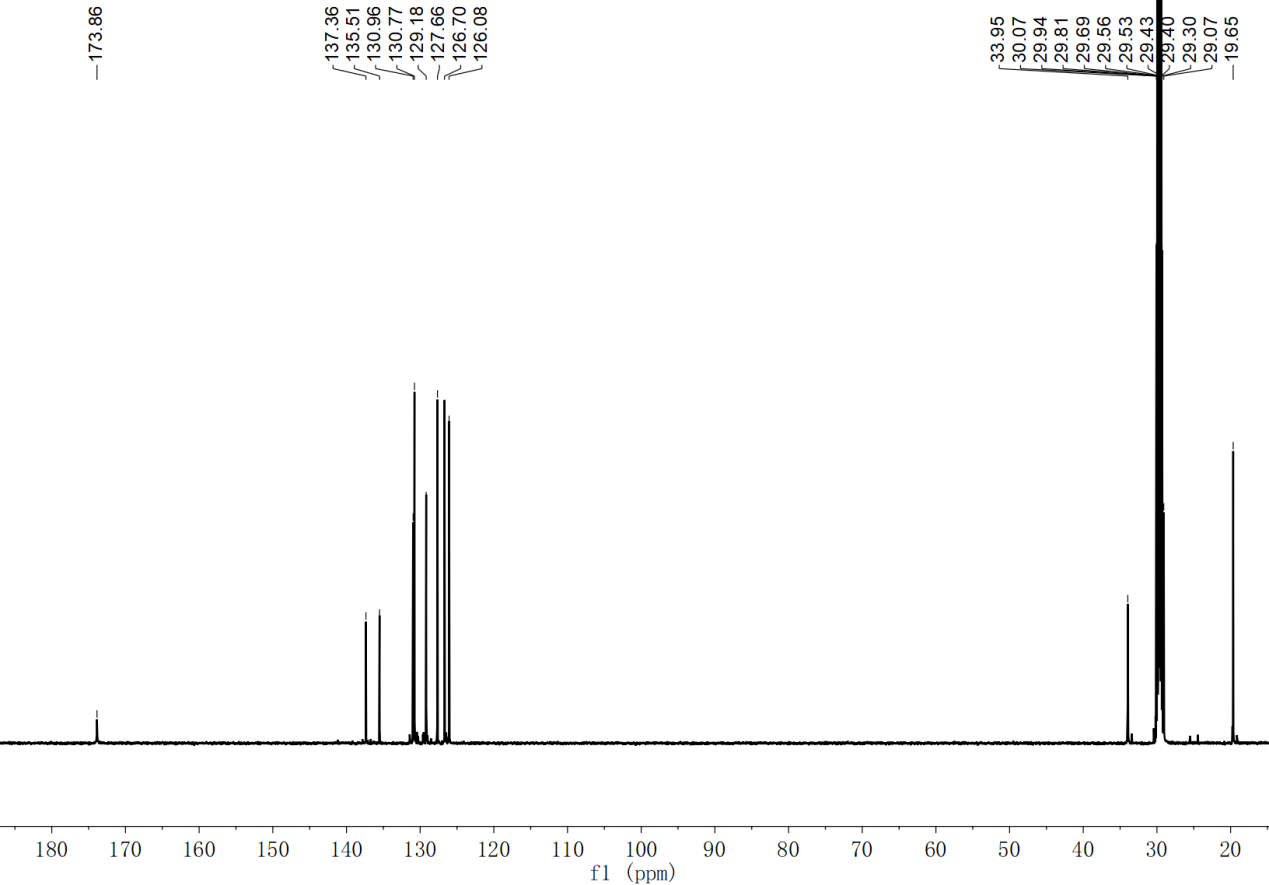


Figure S12. ^13^C-NMR spectrum of compound 2 (125 MHz, (CD_3_)_2_CO).

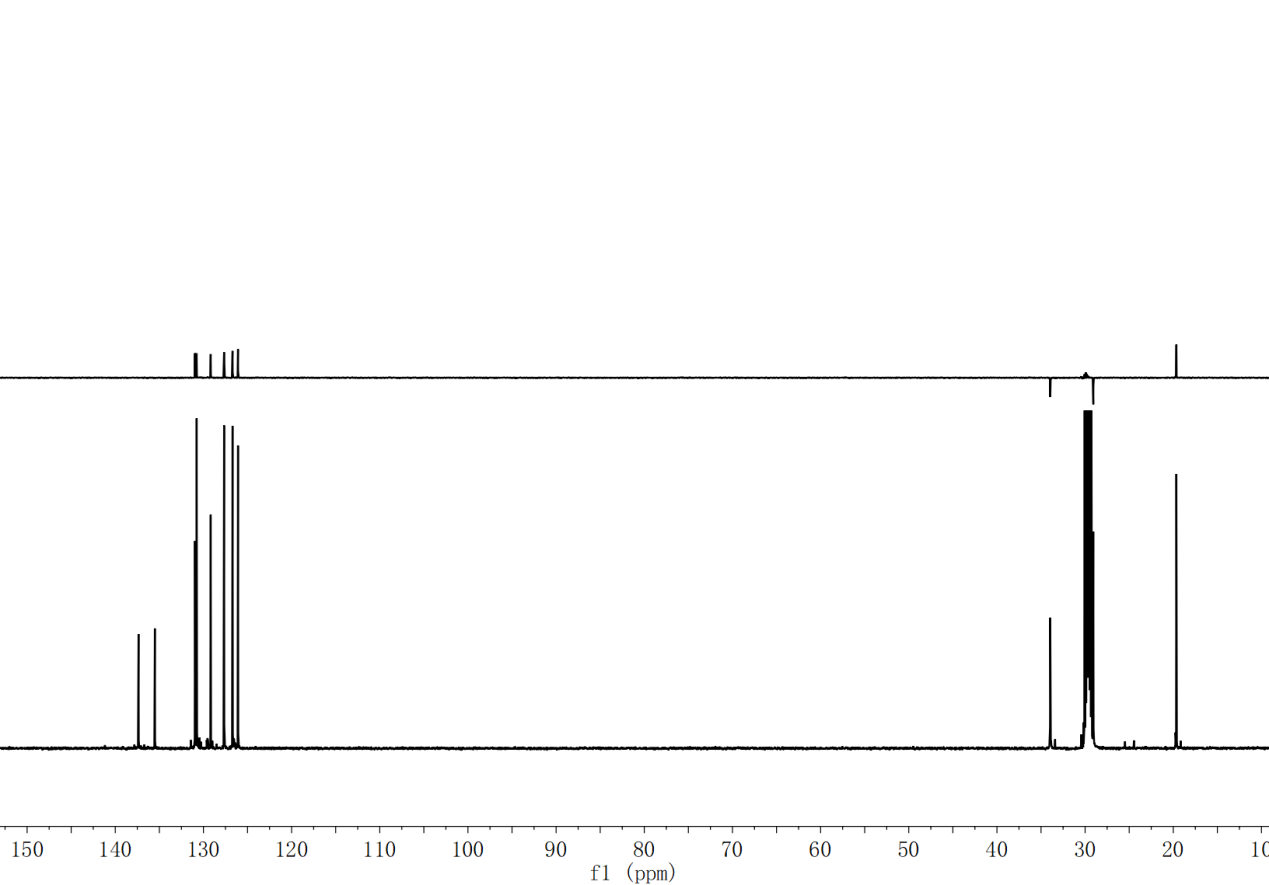


Figure S13. DEPT spectrum of compound 2 (125 MHz, (CD_3_)_2_CO).

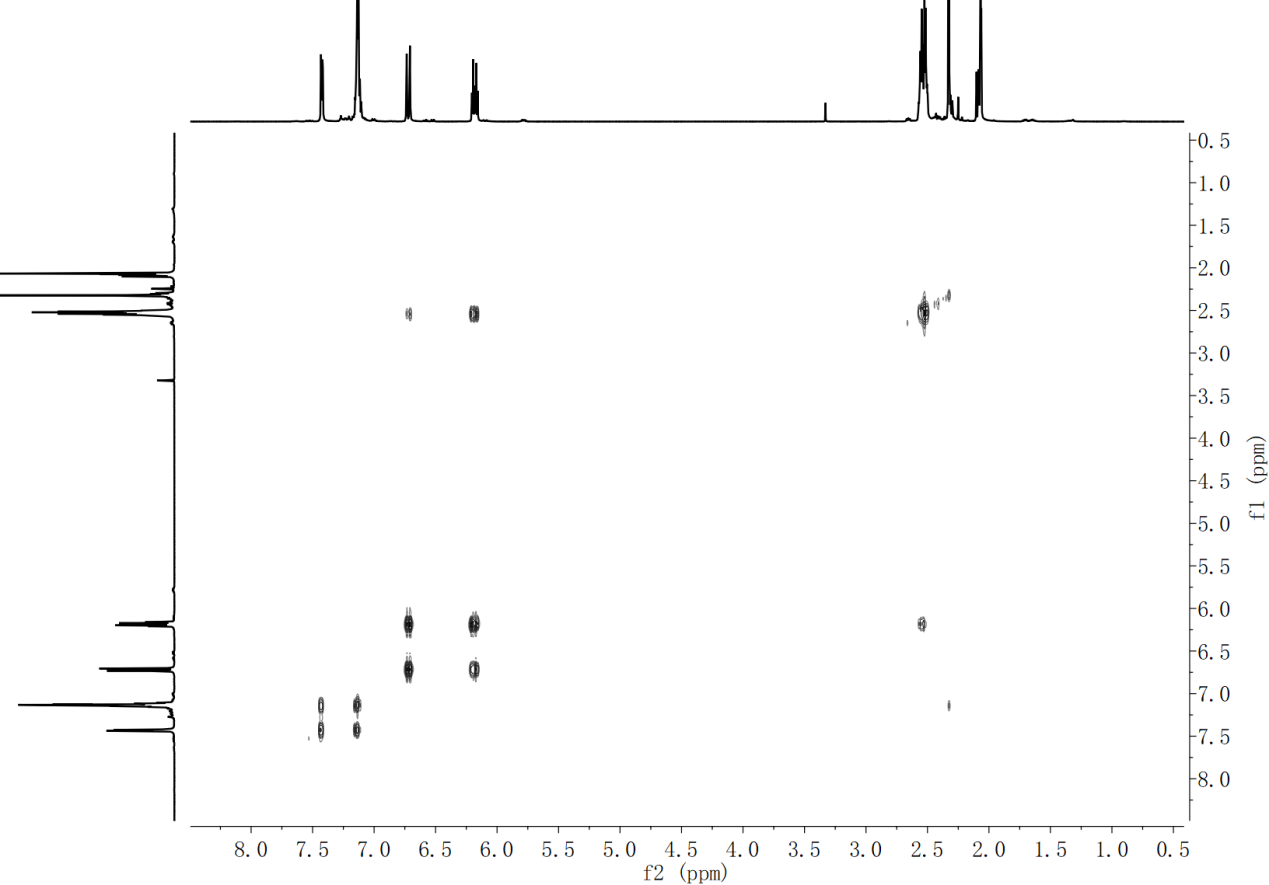


Figure S14. ^1^H-^1^H COSY spectrum of compound 2 ((CD_3_)_2_CO).

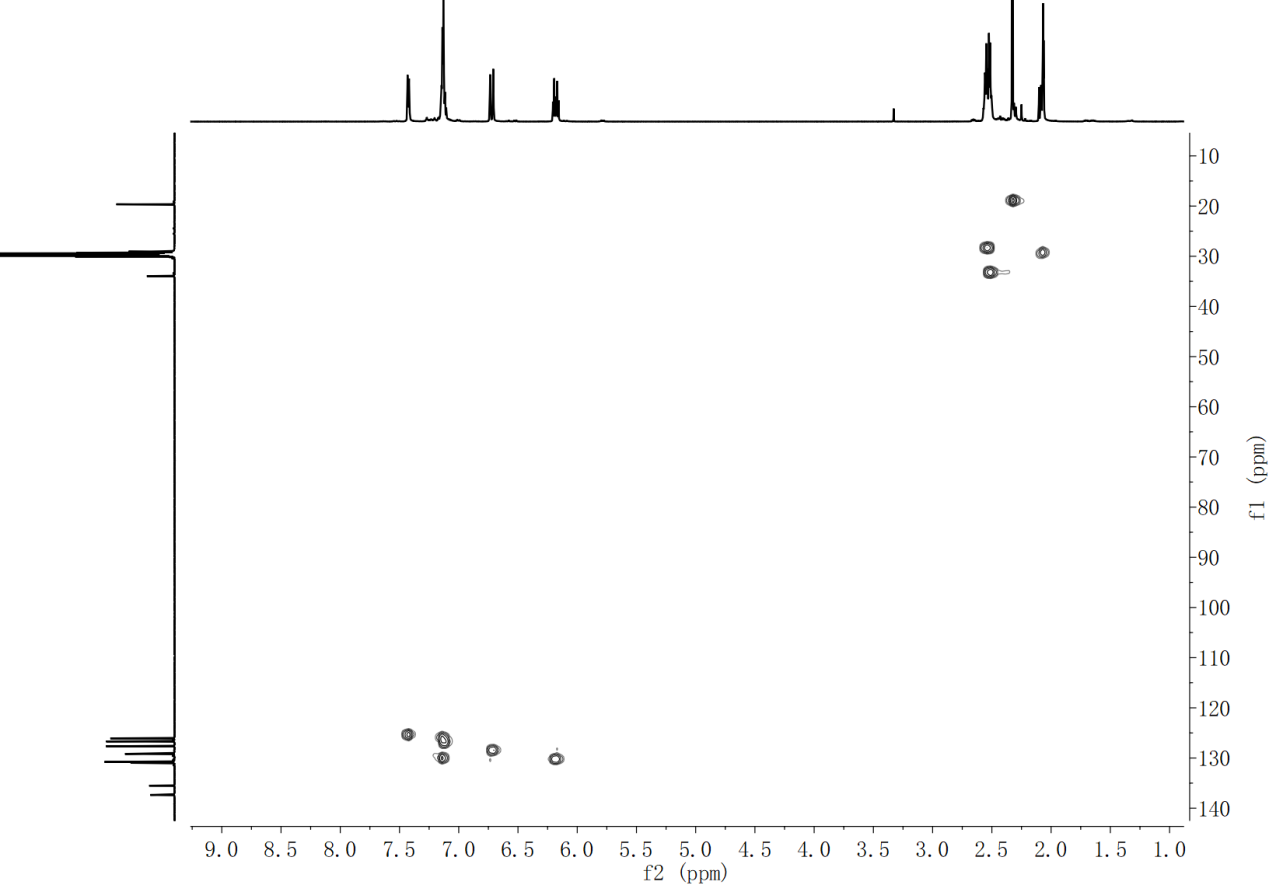


Figure S15. HSQC spectrum of compound 2 ((CD_3_)_2_CO).

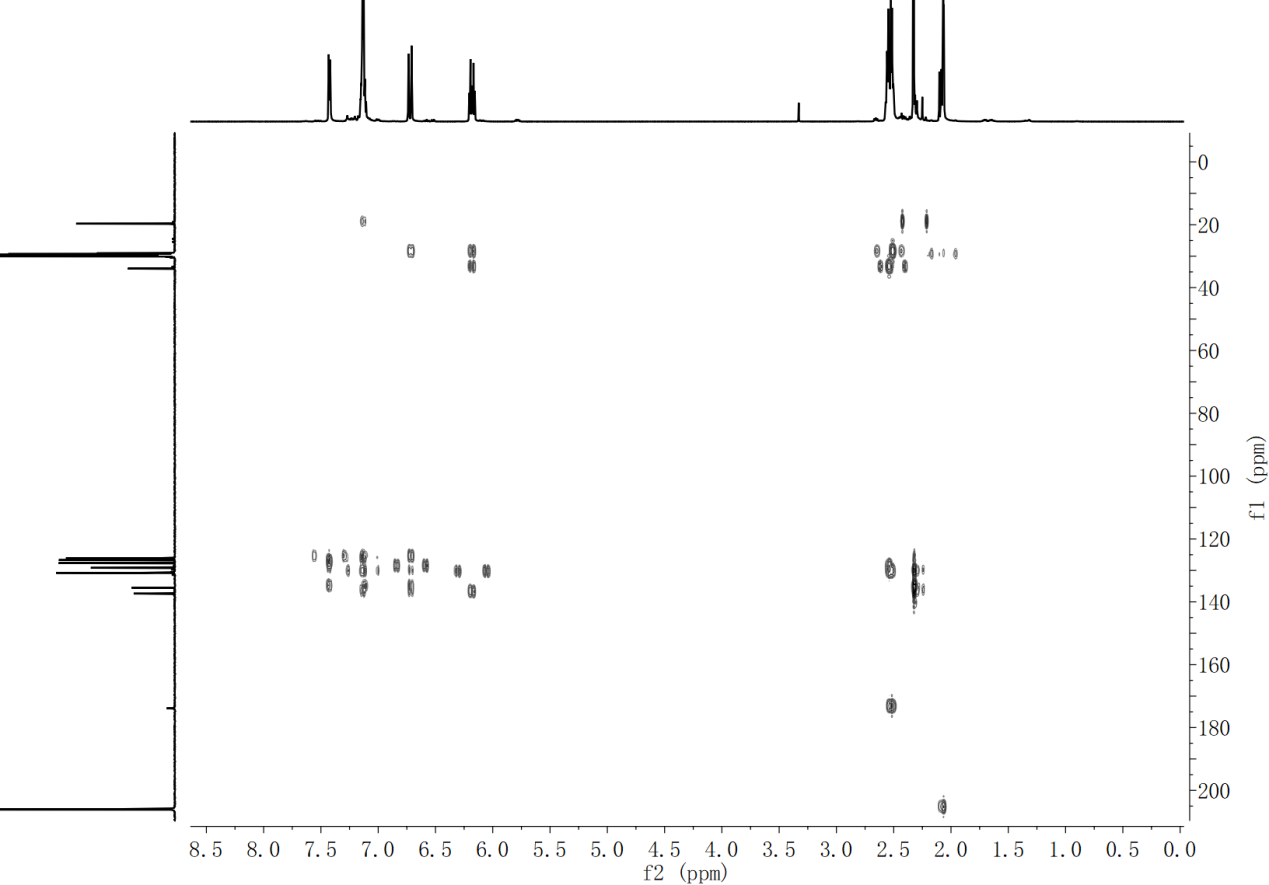


Figure S16. HMBC spectrum of compound 2 ((CD_3_)_2_CO).


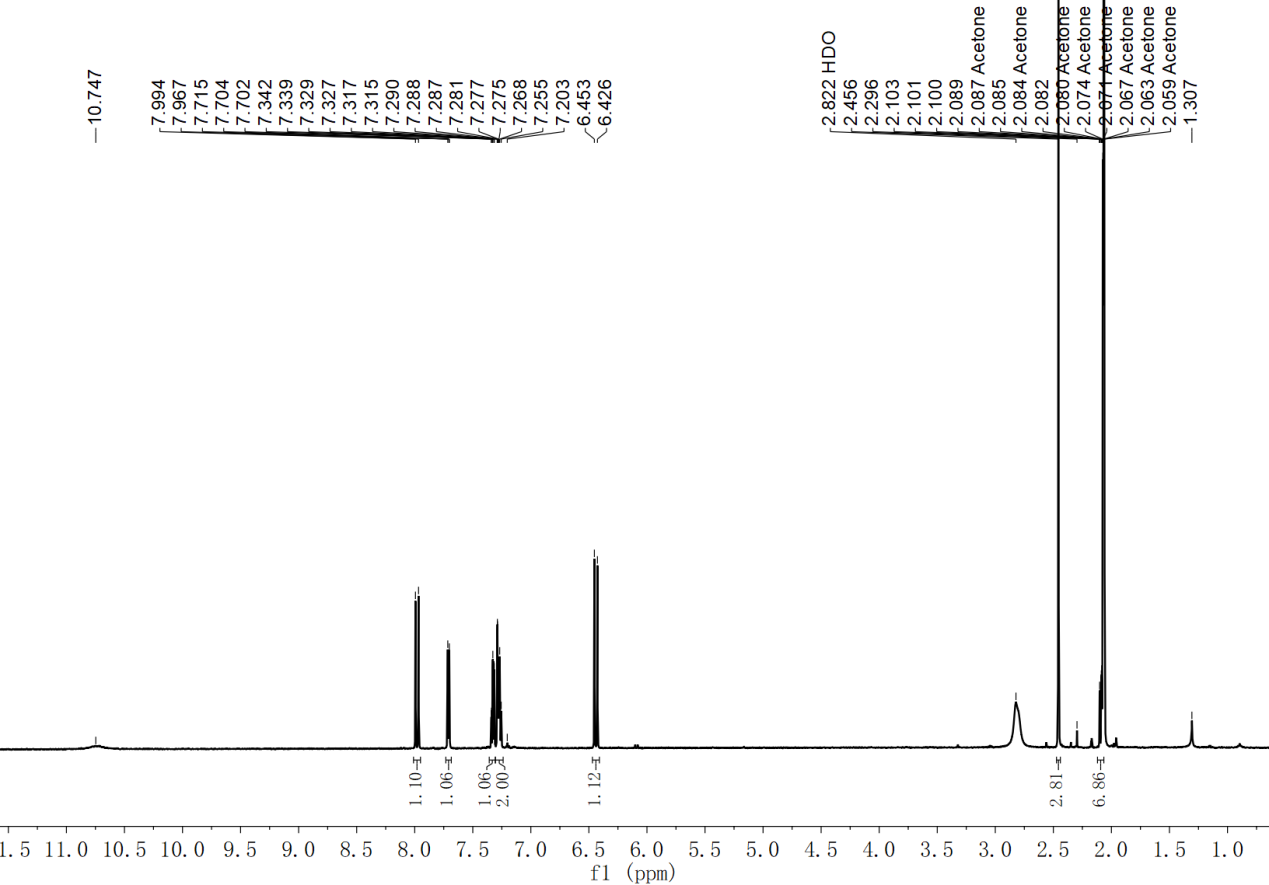


Figure S17. ^1^H-NMR spectrum of compound 3 (500 MHz, (CD_3_)_2_CO).

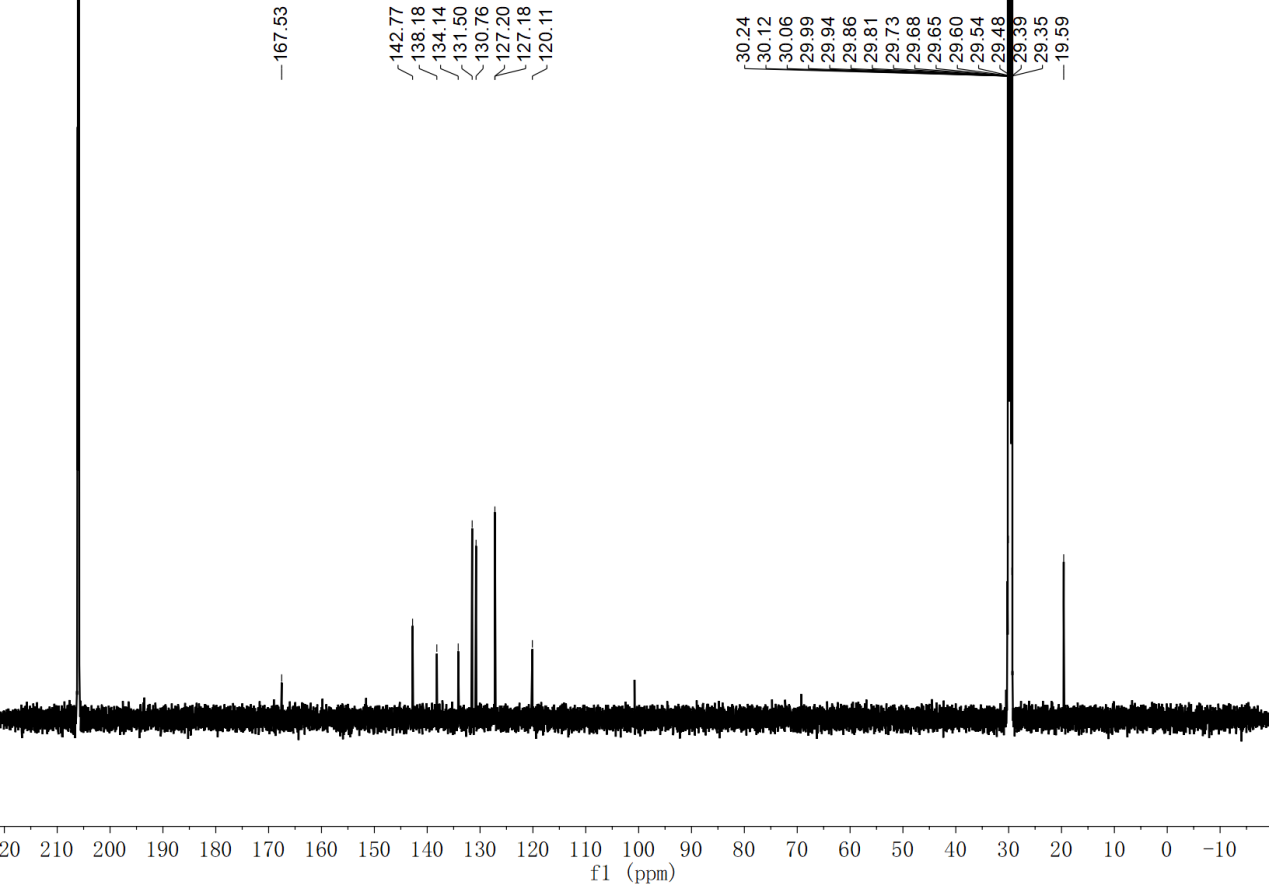
Figure S18. ^13^C-NMR spectrum of compound 3 (125 MHz, (CD_3_)_2_CO).

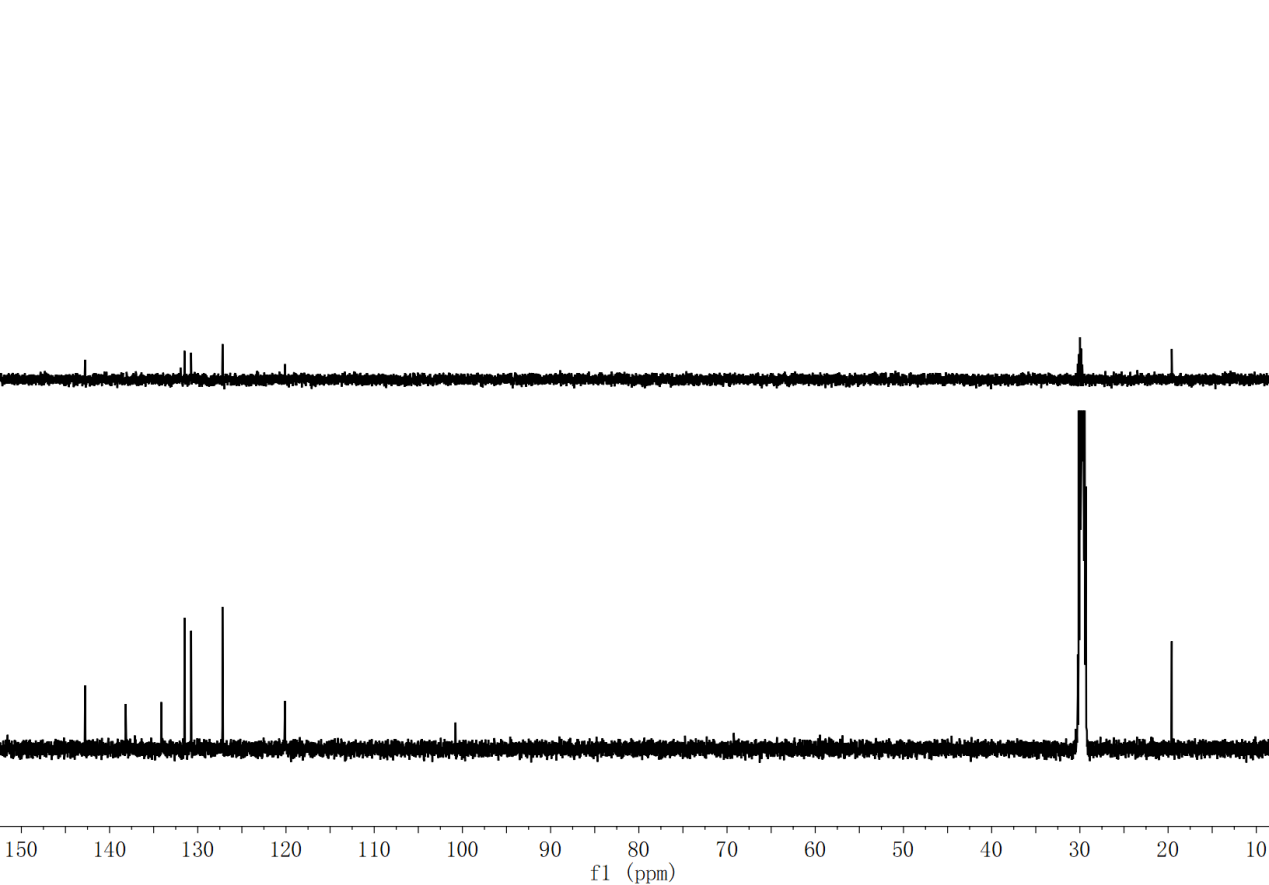


Figure S19. DEPT spectrum of compound 3 (125 MHz, (CD_3_)_2_CO).

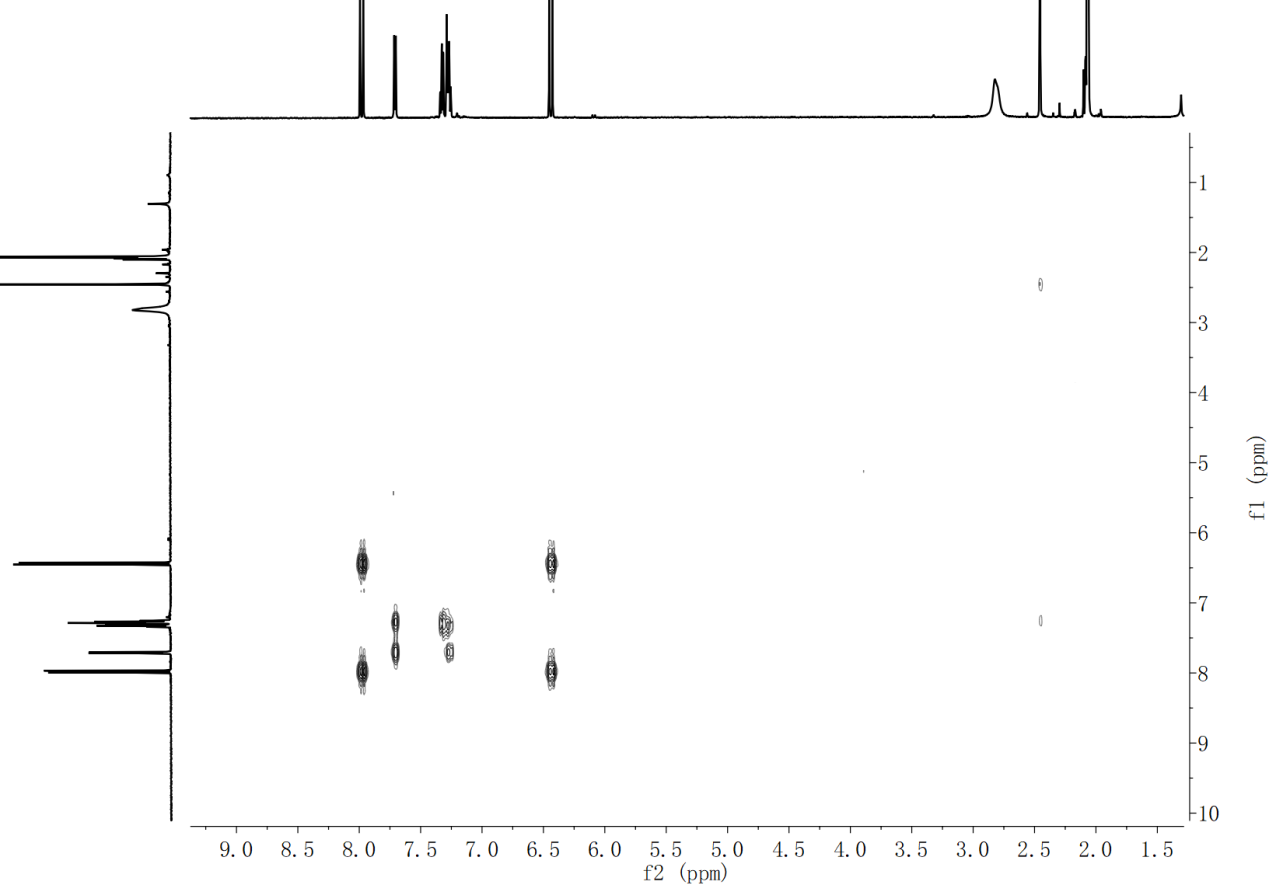


Figure S20. ^1^H-^1^H COSY spectrum of compound 3 ((CD_3_)_2_CO).

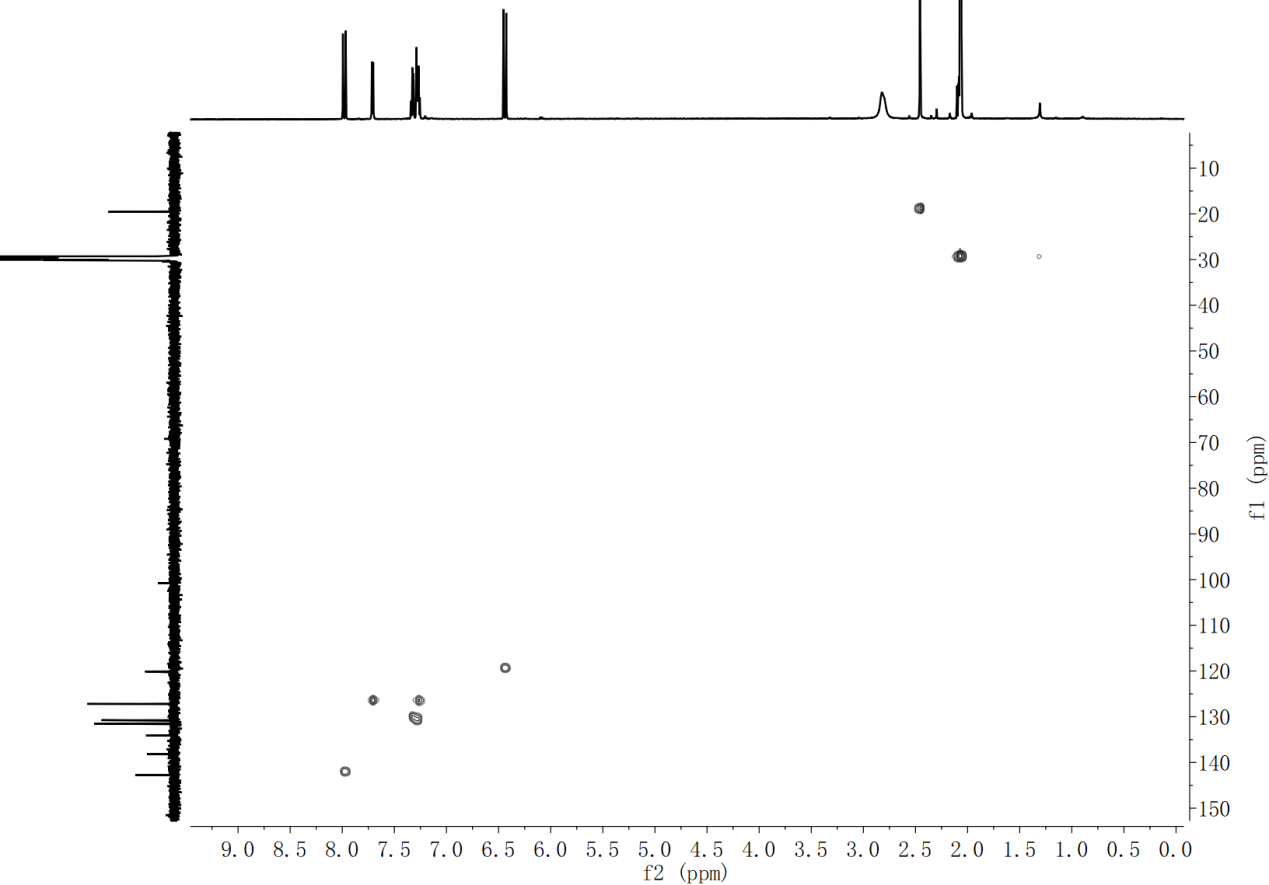
Figure S21. HSQC spectrum of compound 3 ((CD_3_)_2_CO).

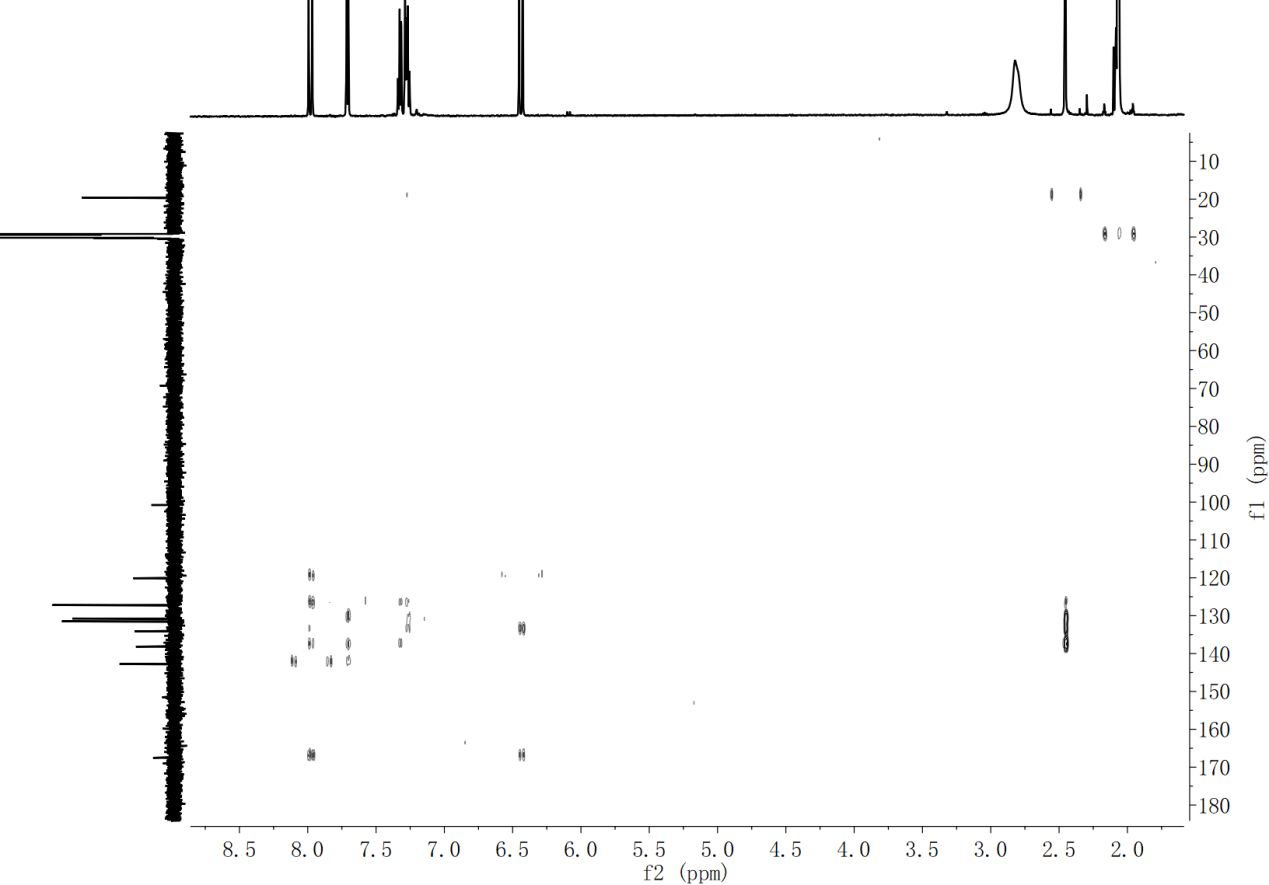


Figure S22. HMBC spectrum of compound 3 ((CD_3_)_2_CO).


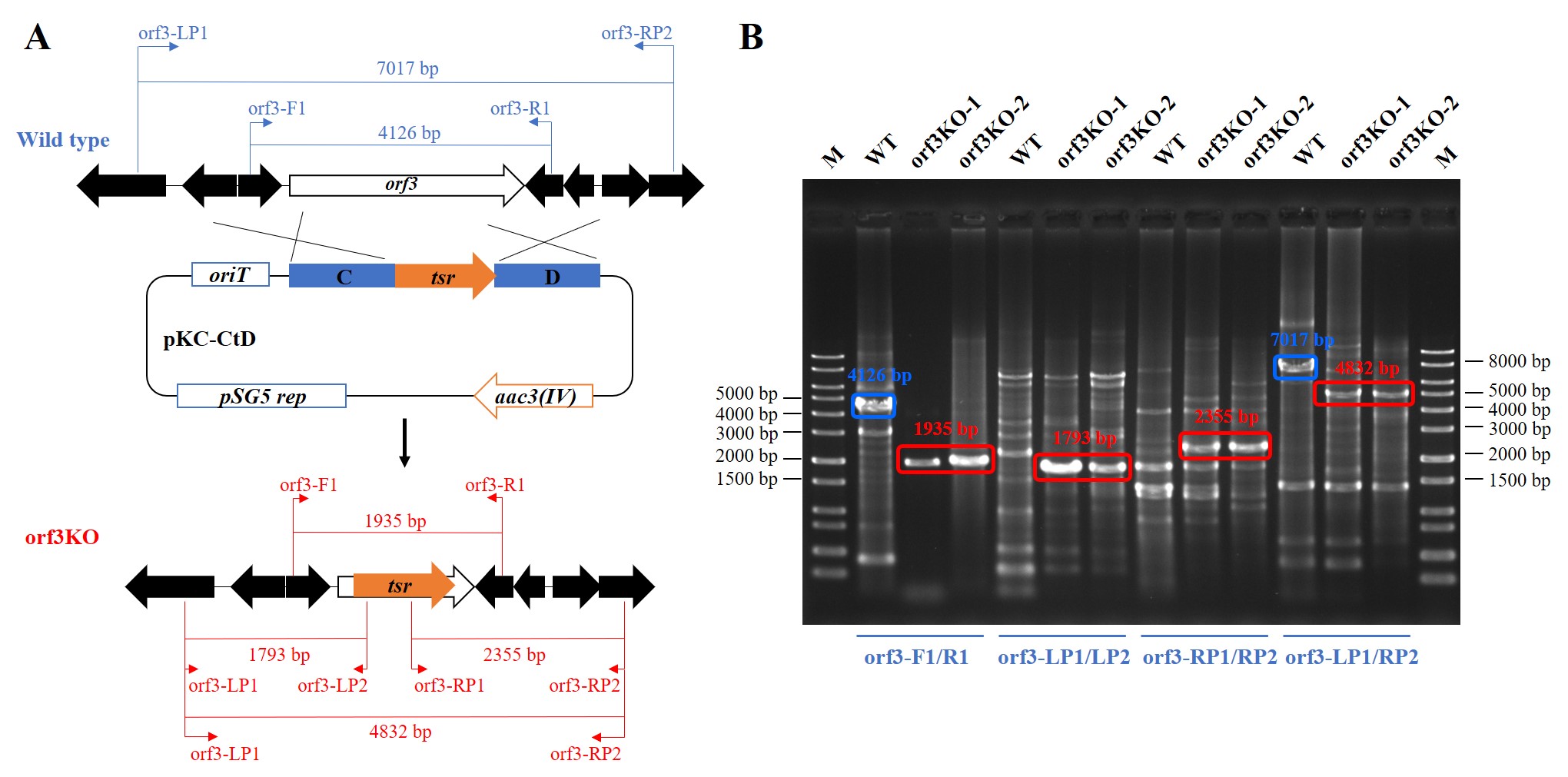


# Figure S23. Disruption of *orf3* gene in *S. globisporus* C-1027.

**A)** Schematic representation for disruption of *orf3* gene. *tsr*, thiostrepton resistance gene. **B)** PCR analysis of the control strain (WT, *S. globisporus* C-1027) and the orf3KO (*orf3* knockout) mutant were carried out with primers listed in Table S2. M: DNA molecular ladder. The size of the predicted and observed PCR fragments was shown by rectangular box.


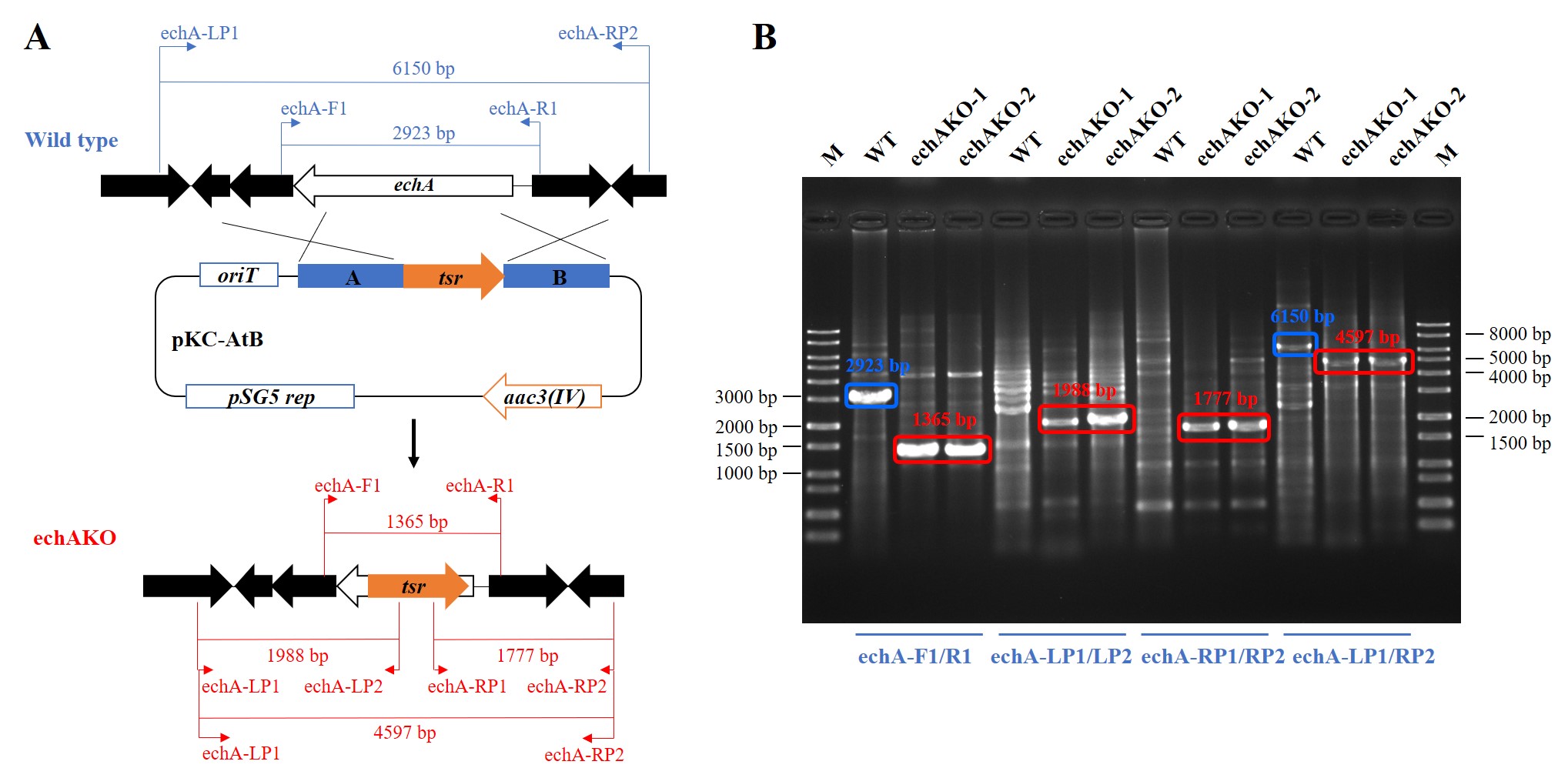


# Figure S24. Disruption of *echA* gene in *S. globisporus* C-1027.

**A)** Schematic representation for disruption of *echA* gene. *tsr*, thiostrepton resistance gene. **B)** PCR analysis of the control strain (WT, *S. globisporus* C-1027) and the echAKO (*echA* knockout) mutant were carried out with primers listed in Table S2. M: DNA molecular ladder. The size of the predicted and observed PCR fragments was shown by rectangular box.


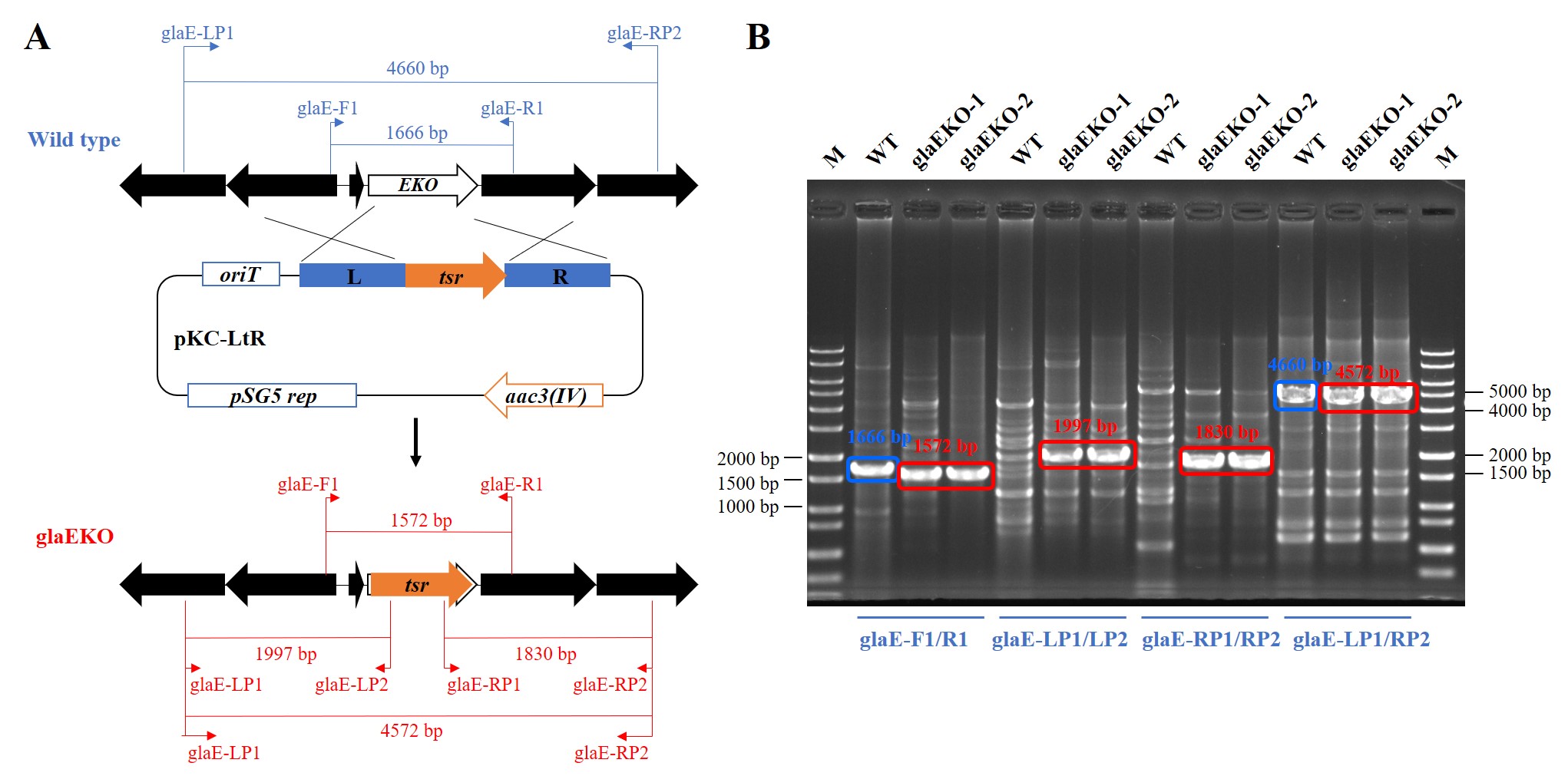


# Figure S25. Disruption of *glaE* gene in *S. globisporus* C-1027.

**A)** Schematic representation for disruption of *glaE* gene. *tsr*, thiostrepton resistance gene. **B)** PCR analysis of the control strain (WT, *S. globisporus* C-1027) and the glaEKO (*glaE* knockout) mutant were carried out with primers listed in Table S2. M: DNA molecular ladder. The size of the predicted and observed PCR fragments was shown by rectangular box.


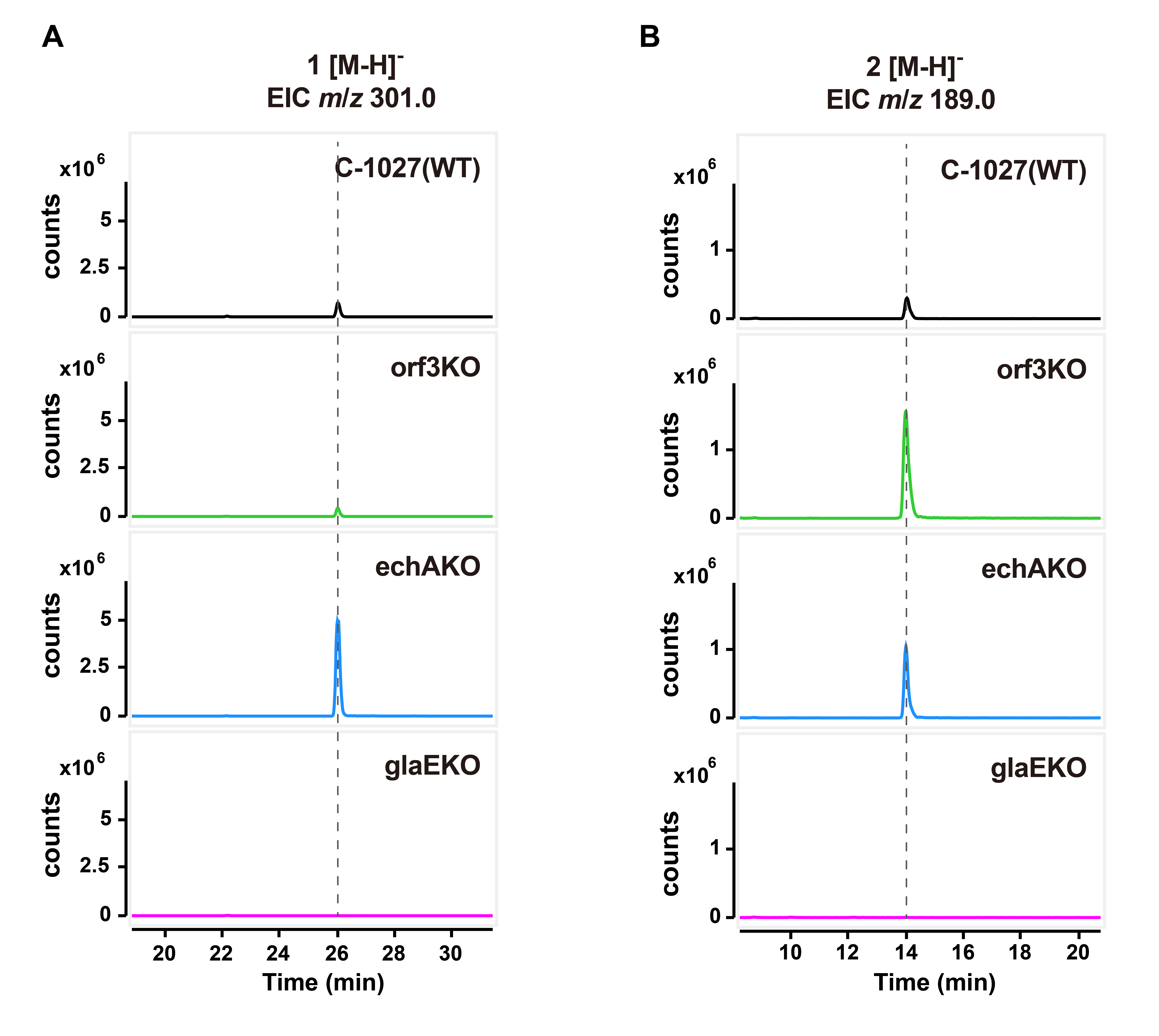


# Figure S26. EIC traces of three knockout mutants.

LC-MS analyses monitor the production of compounds **1** (**A**) and **2** (**B**) in wild-type (WT, C-1027) and knockout mutants. EIC, Extracted ion chromatograms.

**

**

# Figure S27. The phylogenetic trees for KSs and CLFs.

**A)** The phylogenetic tree for *β*-ketoacyl synthases (KSs). **B)** The phylogenetic tree for chain length factors (CLFs). The multiple alignments were done with 10-gap setting and 0.2-gap extension in ClustalW. The trees were constructed by Neighbor-joining method with bootstrap analysis of 1,000 replications in MEGA-X. Bootstrap values given in percentages were shown at the nodes. The KSs and CLFs in *gla* cluster were highlighted in red.

**
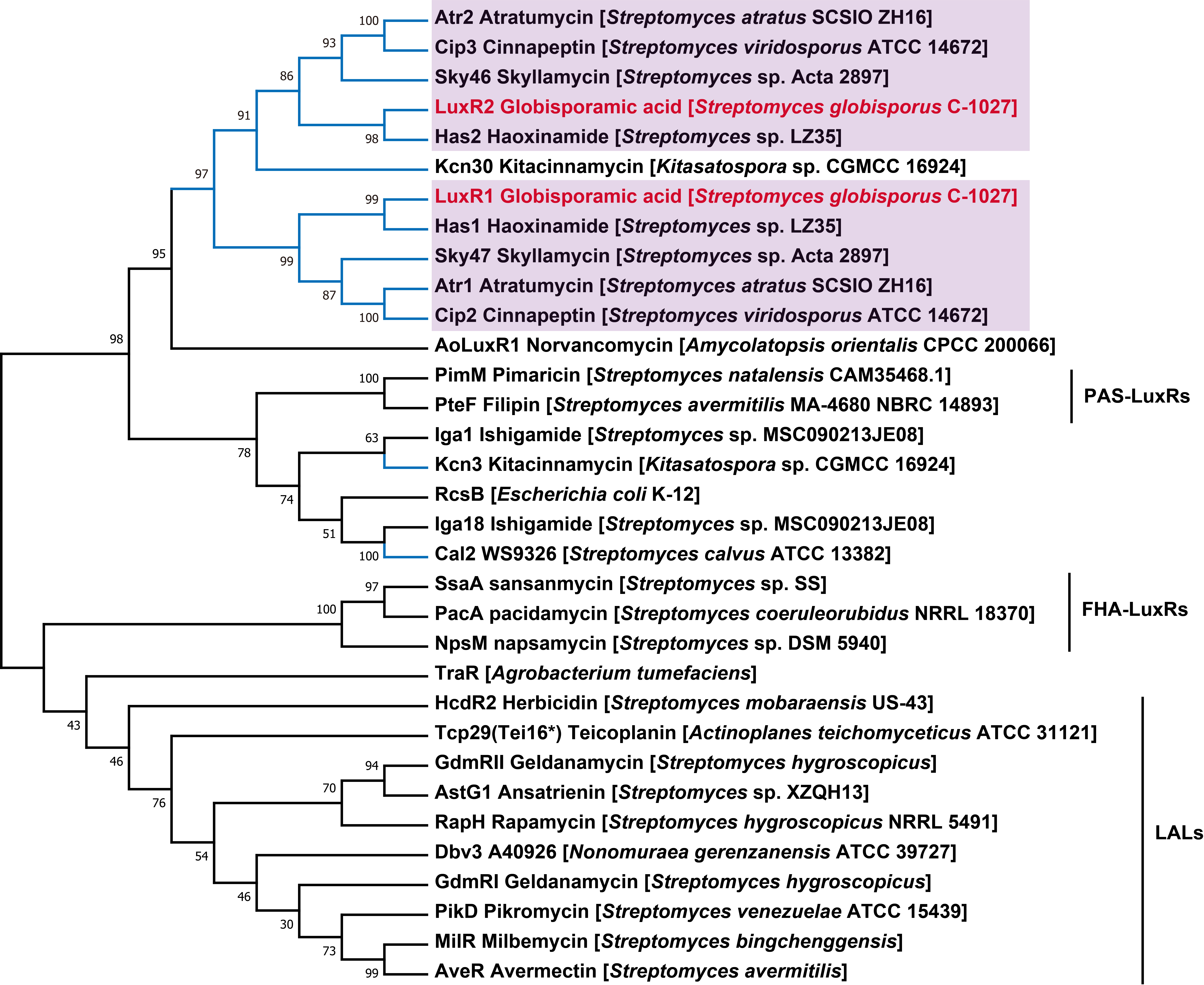
**

# Figure S28. The phylogenetic tree for LuxR-family regulators.

The multiple alignment was done with 10-gap setting and 0.2-gap extension in ClustalW. The tree was constructed by Neighbor-joining method with bootstrap analysis of 1,000 replications in MEGA-X. Bootstrap values given in percentages were shown at the nodes. The LuxR1 and LuxR2 in *gla* cluster were highlighted in red. The putative LuxRs in cinnamoyl moiety-containing product BGCs were labeled by blue. Two adjacent LuxRs in the same cluster were highlighted in purple shade. FHA-LuxRs, fork head-associated (FHA) domain-containing LuxRs; PAS-LuxRs, PAS domain-containing LuxRs; LALs, characterized large ATP-binding regulators of the LuxR family.

# References

Bierman, M., Logan, R., O'Brien, K., Seno, E.T., Rao, R.N., and Schoner, B.E. (1992). Plasmid cloning vectors for the conjugal transfer of DNA from *Escherichia coli* to *Streptomyces* spp. *Gene* 116(1)**,** 43-49. doi: 10.1016/0378-1119(92)90627-2.

Gunstone, F.D., Polard, M.R., Scrimgeour, C.M., and Vedanayagam, H.S. (1977). Fatty acids. Part 50. 13C nuclear magnetic resonance studies of olefinic fatty acids and esters. *Chem Phys Lipids* 18(1)**,** 115-129. doi: 10.1016/0009-3084(77)90031-7.

Hong, B., Phornphisutthimas, S., Tilley, E., Baumberg, S., and McDowall, K.J. (2007). Streptomycin production by *Streptomyces griseus* can be modulated by a mechanism not associated with change in the *adpA* component of the A-factor cascade. *Biotechnol Lett* 29(1)**,** 57-64. doi: 10.1007/s10529-006-9216-2.

Hu, J.L., Xue, Y.C., Xie, M.Y., Zhang, R., Otani, T., Minami, Y., et al. (1988). A new macromolecular antitumor antibiotic, C-1027. I. Discovery, taxonomy of producing organism, fermentation and biological activity. *J Antibiot (Tokyo)* 41(11)**,** 1575-1579. doi: 10.7164/antibiotics.41.1575.

Kieser, T., Bibb, M., Buttner, M., Chater, K., and Hopwood, D. (2000). *Pratical Streptomyces Gnetics.* Norwich: The John Innes Foundation.
